# Statistical Mechanics Provides Novel Insights into Microtubule Stability and Mechanism of Shrinkage

**DOI:** 10.1371/journal.pcbi.1004099

**Published:** 2015-02-18

**Authors:** Ishutesh Jain, Mandar M. Inamdar, Ranjith Padinhateeri

**Affiliations:** 1 Department of Biosciences and Bioengineering, Indian Institute of Technology Bombay, Mumbai, India; 2 Department of Civil Engineering, Indian Institute of Technology Bombay, Mumbai, India; University of Michigan, United States of America

## Abstract

Microtubules are nano-machines that grow and shrink stochastically, making use of the coupling between chemical kinetics and mechanics of its constituent protofilaments (PFs). We investigate the stability and shrinkage of microtubules taking into account inter-protofilament interactions and bending interactions of intrinsically curved PFs. Computing the free energy as a function of PF tip position, we show that the competition between curvature energy, inter-PF interaction energy and entropy leads to a rich landscape with a series of minima that repeat over a length-scale determined by the intrinsic curvature. Computing Langevin dynamics of the tip through the landscape and accounting for depolymerization, we calculate the average unzippering and shrinkage velocities of GDP protofilaments and compare them with the experimentally known results. Our analysis predicts that the strength of the inter-PF interaction ($E_{m}^{s}$) has to be comparable to the strength of the curvature energy ($E_{m}^{b}$) such that $E_{m}^{s}-E_{m}^{b}\approx 1k_{\text{B}}T$, and questions the prevalent notion that unzippering results from the domination of bending energy of curved GDP PFs. Our work demonstrates how the shape of the free energy landscape is crucial in explaining the mechanism of MT shrinkage where the unzippered PFs will fluctuate in a set of partially peeled off states and subunit dissociation will reduce the length.

## Author Summary

Microtubules are cylindrical machines inside biological cells, and are crucial for many functions such as chromosome segregation, intra-cellular transport, and cell motility. They are made of 13 elastic filaments (protofilaments) that can be either in a straight or in a curved conformation depending on the chemical state of the constituent tubulin molecules. The interplay between these two conformations help microtubules to display a fascinating phenomenon known as “dynamic instability,” in which the microtubule steadily self-assembles and catastrophically disassembles in a seemingly random process. During the disassembly, the protofilaments are known to curve out forming ram’s horn-like structures. Scientists have been trying to understand how the laws of mechanics and statistical thermodynamics determine the complex behavior of microtubules. In this paper, we investigate how the chemical bonding between protofilaments, bending elasticity of protofilaments, and thermal forces determine the speed of the disassembly of the microtubule cylinder. We show that the current notion of a bending-elasticity dominated disassembly will lead to a paradox; resolving the paradox, we argue that the disassembly is a result of an intricate molecular orchestration in which the thermal and curvature energies of protofilaments compete with inter-protofilament bonding energy leading to curving out and depolymerization.

## Introduction

Microtubules (MT) are a unique kind of supramolecular structures that are integral to a number of cellular processes, such as chromosome segregation, cell motility, intracellular transport and organization [1–4]. They are always in a dynamic state, displaying a phenomenon known as dynamic instability, characterized by cycles of sustained growth and rapid shrinkage [5, 6]. Dynamic instability of MTs is essential for many of the cellular functions, and the cells actively regulate microtubule growth and shrinkage during many of these crucial cellular processes [6–16]. Thus, understanding the mechanism of dynamic instability and its regulation is essential in understanding cell function, and remains a topic of active research.

**Figure 1:**
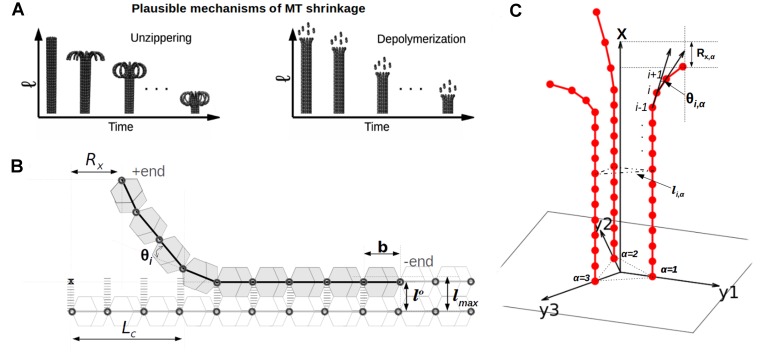
Model. (A) Unzippering leads to shrinkage; hence, the unzippering velocity cannot be much larger than the experimentally observed shrinkage velocity. If depolymerization is much faster than unzippering, no ram’s horns will be seen (see text). (B) Schematic depiction of single PF interacting with a rigid neighbor: PF is made of *N* subunits, each of length *b*. *θ*_*i*_ is the relative angle between two subunits, specified in the bending energy. Each subunit interacts with its neighbor in the adjacent PF via a breakable Hookean spring. This lateral spring-like bond has an equilibrium length of *l* ^*o*^ and is considered to be “broken” if the extension of the spring is larger than *l*_max_. *L*_*c*_ is the peeled-off length—length of the region in which the lateral-bonds are broken. *R*_*x*_ represents the *x* position of the tip of the semiflexible protofilament. (C) Schematic representation of the multi-protofilament model: The microtubule is made of three protofilaments (*α* is the protofilament index); each protofilament is made of *N* subunits, each of length *b* (*i* is the subunit index). Each protofilament interacts with two neighbors via the lateral interaction given by Eq. 6. Each protofilament in this model can bend and fluctuate in their respective planes, shown by *x*−*y* ^*α*^(*α* ∈ {1,2,3}).

A microtubule is a tube-like structure made of 13 protofilaments (PFs) that are formed by polymerizing GTP tubulin (T) subunits. These GTP-protofilaments are thought to be straight and form inter-protofilament (lateral) bonds with neighbors, stabilizing the tubular structure [17, 18]. However, the GTP-subunits on the filament hydrolyze into GDP (D) subunits and form GDP-rich protofilaments that prefer to be in a curved state [19, 20]. This induction of curvature leads to a “power struggle” between inter-protofilament interactions and the bending elasticity of intrinsically curved GDP protofilaments [21–23]. It has been been experimentally observed that a GDP-rich microtubule is unstable, forms curved “ram’s horn”-like structures, and shrinks rapidly [19–20, 24]. This observation has led to the notion that for microtubules composed of GDP protofilaments, bending elasticity dominates over lateral interactions leading to instability and catastrophe [21–22, 25]. Catastrophe is a crucial event thought to be triggered by the mechanical instability of MT, in which MT switches from a growing state to a rapidly shrinking state. This shrinkage during catastrophe can happen due to two events—unzippering (peeling off) of protofilaments as a result of the mechanical power struggle, or the kinetic event of depolymerization (dissociation) of subunits (see Fig. 1(A)). However, since the protofilament unzippering will, in itself, result in experimentally observable microtubule “length change” (shrinkage), there exists a clear constraint that the average unzippering velocity of protofilaments cannot be much greater than the experimentally observed speed of MT length change (shrinkage velocity). On the other hand, unzippering velocity cannot be much lesser than the known shrinkage velocity either—that is, the shrinkage cannot happen only via depolymerization—as this would contradict the fact that curved protofilaments (ram’s horns) are observed experimentally. This suggests that protofilament unzippering induced by mechanical interactions must have a timescale that is roughly comparable to the known timescale of microtubule shrinkage. Therefore, to understand the microtubule dynamics adequately, it is essential to connect the kinetics of MT shrinkage with the kinetics of protofilament unzippering.

It is common to describe complete non-equilibrium dynamics of microtubules using four kinetic parameters—growth velocity, shrinkage velocity, catastrophe frequency, and rescue frequency [6, 26, 27]. However, these parameters themselves crucially depend on the mechano-chemical state of the protofilaments, inter-PF interactions and the thermal environment. To the best of our knowledge, there exists no work in the literature that investigates how the mechanical interactions, in the presence of thermal fluctuations lead to the deduction of any of these four parameters, and reveal a mechanism of the underlying process. There have been a number of papers that investigate how the mechanical changes emerge from the underlying structural rearrangement using molecular dynamics and coarse-grained simulations [28–30]. However, such molecular simulations can probe the behavior of only a few tubulin subunits (a length scale much smaller than the scale of a protofilament) and can only simulate for a timescale that is much shorter than the typical shrinkage timescale. Therefore different groups have been employing models that are further coarse-grained, focusing on the energetics of protofilaments [21–23, 31–34]. Even though these models find that the mechanical aspects of MT interactions are crucial [21–23, 31–35] for occurrence of rams’ horns, none of these models, except ref.[35], account for protofilament thermal fluctuations, which are critical in formation as well as in overcoming of local energy barriers that greatly influence the overall PF unzippering dynamics. Also, none of the existing models have systematically examined the velocity of protofilament unzippering, dissociation of tubulin subunits and their connection with the experimentally observed velocity of MT shrinkage. In short, it is not known how inter-PF, bending interactions and entropy control the unzippering process; neither is it known how the interplay between unzippering and dissociation of subunits influence shrinkage dynamics.

In this paper, we demonstrate how inter-PF interactions, PF bending, PF configurational entropy, and dissociation of subunits determine one of the four parameters—the shrinkage velocity of microtubules. This exercise also leads to a number of counter-intuitive testable predictions. We study a micro-mechanical model of MT protofilaments that takes into account inter-protofilament and bending interactions in the presence of thermal fluctuations. Using Monte Carlo simulations, we construct the free energy landscape for MT protofilaments. We show that the competition between bending energy, inter-protofilament (lateral) interaction energy and entropy leads to a rich free energy landscape with multiple minima that repeat over the length scale of the intrinsic curvature. Considering unzippering of PFs as movement through the free energy landscape and dissociation of subunits as stochastic jumps, we simulate the complete shrinkage dynamics of MT using Langevin simulations. We find that the minima and the shape of the free energy landscape can slow down the unzippering GDP protofilaments by temporarily restricting them in partially peeled off states, and can potentially help the depolymerization catch up with the peeling-off. By comparing the simulated unzippering velocity and shrinkage velocity with the experimentally known value, our theory makes an important conclusion and a set of predictions: (1) Unlike the prevalent picture, the strength of the bending energy ($E_{m}^{b}$) of GDP protofilaments cannot dominate over the strength of the lateral interaction energy ($E_{m}^{s}$); $E_{m}^{b}\gg E_{m}^{s}$ will lead to a paradox, in which one will not be able to simultaneously explain the observed shrinkage velocity and structural features (like a ram’s horn) associated with MT shrinkage. (2) The paradox can only be resolved if $E_{m}^{s}-E_{m}^{b}\approx k_{\text{B}}T$; therefore, this has to be the relative strength of the MT interaction parameters. (3) In the sensible parameter regime ($E_{m}^{s}-E_{m}^{b}\approx k_{\text{B}}T$), unzippering and subunit dissociation work hand in hand, leading to MT shrinkage. The unzippering here is dominated by thermal fluctuations. (4) Our theory also predicts measurable quantities such as the lifetime of ram’s horn curls and the number of unzippering events. (5) We show that even a single GTP cap per protofilament can delay the unzippering process beyond polymerization timescales. We also study a multi-protofilament model and examine how independent unzippering fluctuations of each protofilament would affect MT stability. For the quantities computed in this paper, we find that the results from the multi-protofilament model are comparable to the results of the single-protofilament model.

## Model

In our model, we take into account three important factors that determine microtubule stability: (a) inter-protofilament interaction—interaction between neighboring protofilaments via lateral bonds (or lateral interaction), (b) bending elasticity of protofilaments—interaction between neighboring molecular units in a protofilament, that leads to certain preferred bond angles between them (see details below), and (c) thermal fluctuations. The first interaction stems from the fact that each protofilament, in a microtubule, interacts with two of its neighbors. The second interaction accounts for the known fact that GDP-rich protofilaments prefer to be in an intrinsically curved state, while GTP-rich protofilaments are found to be straight. Given that these two interactions are competing with each other, the third factor, thermal fluctuations, can be of great relevance in tilting the balance in favor of one or the other. This aspect of three-way competition will be significant to understand the results of this paper.

We study statistical mechanics of microtubules using two different versions of the model: (i) a single semiflexible, thermally fluctuating, protofilament interacting with a rigid neighbor and (ii) a multiprotofilament model in which each protofilament interacts with two neighboring protofilaments—each protofilament is bendable and can independently fluctuate thermally. The aim of the first (and simpler) version of the model is to learn the essential physics behind the complex biological problem—to understand how the competition between lateral interaction energy, curvature energy and entropy would play out and influence microtubule stability and kinetics. The aim of the second version of the model is to examine the role of fluctuating neighbors and to check whether the physical insights we gained from the simpler model would hold in a more complex situation.

### Single protofilament interacting with a rigid neighbor

We model a toy version of microtubule as a set of two interacting protofilaments: each protofilament is a chain made of *N* rod-like discrete subunits of fixed length *b* (see Fig. 1(B)). We assume that one protofilament is semiflexible (bendable) while the neighboring protofilament is infinitely rigid and is permanently fixed in space. Each subunit in a protofilament interacts laterally with its subunit pair in the neighboring protofilament via a breakable Hookean spring, energy of which is given by

$$E_{i}^{s}\;=\;\frac{k^{s}}{2}{\left(l_{i}-l^{o}\right)}^{2},\;\text{when}\;l_{i}<l_{\text{max}}$$ (1)

$$E_{i}^{s}\;=\;\frac{k^{s}}{2}{\left(l_{\text{max}}-l^{o}\right)}^{2},\;\text{when}\;l_{i}\geq l_{\text{max}}$$ (2)

where *k* ^*s*^ is the spring constant of an individual lateral bond, *l*_*i*_ is the distance between *i* ^th^ subunit of the semiflexible protofilament with its fixed lateral neighbor, *l* ^*o*^ is the equilibrium length of the bond and *l*_max_ is the length beyond which the lateral spring bond is “broken.” To quantify the strength of the lateral interaction, we define a constant $E_{m}^{s}=\frac{1}{2}k^{s}{\left(l_{\text{max}}-l^{o}\right)}^{2}$, which is essentially the maximum energy of tubulin-tubulin lateral bond.

The bendable protofilament is modeled as a discrete worm-like chain with a given intrinsic curvature [36, 37]. The bending energy of the longitudinal bond (also known as curvature energy) to maintain a relative angle of *θ*_*i*_ between adjacent subunits *i* and *i*+1 is given by

$$E_{i}^{b}=k^{b}(1-\cos(\theta_{i}-\theta_{i}^{o})),$$ (3)

where *k* ^*b*^ is the bending stiffness of the longitudinal bond and $\theta_{i}^{o}$ is the preferred intrinsic angle between the longitudinally adjacent subunits that can be different for GTP-subunit($\theta_{i}^{o}=\theta^{T}$) and GDP-subunit ($\theta_{i}^{o}=\theta^{D}$). In the perfectly tubular state, when *θ*_*i*_ = 0, the quantity $k^{b}(1-\cos(\theta^{D}))=E_{m}^{b}$ captures the bending strain energy per subunit stored inside the naturally curved GDP microtubule lattice when it is constrained to be straight. We assume that interactions other than bending and lateral bonding have negligible contribution, and the total energy of the microtubule system is given by

$$E^{\text{tot}}=\sum_{i=1}^{N}\left(E_{i}^{s}+E_{i}^{b}\right),$$ (4)

where the summation spans over all subunits and angles appropriately. Given a particular composition of GDP and GTP subunits, we simulate this microtubule system using the Monte Carlo method (Metropolis algorithm). The Monte Carlo method naturally accounts for the thermal fluctuations of the semiflexible protofilament at a finite temperature *T* (see details below).

Note that, to represent lateral and bending interactions (Eqs. 1, 2, 3), one may use other functional forms that have similar behaviors. For example, the lateral interactions can be, equivalently, represented by a Lennard-Jones potential or Morse potential. Similarly, instead of the cosine functional form in the bending energy, one may use a simple quadratic functional such as $(\kappa /2){\left(\theta_{i}-\theta_{i}^{o}\right)}^{2}$. In Supporting Information (SI), we discuss these different possibilities and their equivalence to the current functional forms that we have used above (see S1 Text).

### Multiple protofilaments interacting with semiflexible neighbors

Even though there are thirteen protofilaments in a microtubule, each protofilament can only interact with two other protofilaments—the two lateral neighbors. Therefore, the simplest multiprotofilament model with realistic features will be a microtubule made of three protofilaments. Also note that three is the minimal number of protofilaments required to form a prismatic beam mimicking a microtubule. Here we construct such a model where a microtubule is depicted as a set of three interacting protofilaments, and each protofilament is a discrete semiflexible (bendable) chain made of *N* subunits (similar to the bendable protofilament above). Each protofilament interacts laterally with two neighboring protofilaments, forming a closed three-dimensional structure, as shown in Fig. 1(C).

The lines connecting the centers of the terminal subunit at the minus end of protofilaments form an equilateral triangle with its centroid at C (see the equilateral triangle at the bottom of Fig. 1(C)). We assume that each protofilament(*α*) can fluctuate only in the plane *x*−*y* ^*α*^(*α* ∈ {1,2,3}), the plane constituted by the central axis of the microtubule (*x*) and the line joining the centroid C with the protofilament *α* (*y* ^*α*^), shown in Fig. 1(C). The total interaction energy of the system is $E^{\text{tot}}=\Sigma_{\alpha =1}^{3}\;\Sigma_{\alpha =1}^{N}(E_{i,\alpha }^{b}+E_{i,\alpha }^{s})$, where $E_{i,\alpha }^{b}$ is the bending energy of *i* ^th^ subunit of the *α* protofilament, and is given by Eq. 3 when *θ*_*i*_ is replaced with *θ*_*i*,*α*_. The energy constituent $E_{i,\alpha }^{s}$ is the lateral interaction energy of the *i* ^th^ unit with the *i* ^th^ subunit on the next protofilament, now calculated as

$$E_{i,\alpha }^{s}\;=\;\frac{1}{2}k^{s}{\left(l_{i,\alpha }-l^{o}\right)}^{2},\;\text{when}\;l_{i,\alpha }<l_{\text{max}}$$ (5)

$$E_{i,\alpha }^{s}\;=\;\frac{1}{2}k^{s}{\left(l_{\text{max}}-l^{o}\right)}^{2},\;\text{when}\;l_{i,\alpha }\geq l_{\text{max}}$$ (6)

where *l*_*i*,*α*_ is the distance between the the *i* ^th^ subunit on protofilament *α* and the *i* ^th^ subunit on the next protofilament in the clockwise direction. As in the previous case, we simulate this microtubule system using the Metropolis Monte Carlo method, as discussed below.

### Computational methods and numerical values of parameters

We simulated these models using the Metropolis Monte Carlo (MC) method (≈ 10^10^MC steps) and obtained various measurable properties at equilibrium [38]. In the simulation, we start with an initial configuration described by the orientations of all subunits (specified by a set of angles {*θ*_*i*_}) and the inter-protofilament distances between neighboring subunits (specified by the distances {*l*_*i*_}). The energy of this initial micro-state is computed as ${ℰ}_{\text{in}}=E^{\text{tot}}$, as described in the model section. To sample through all the micro-states, we choose one tubulin subunit (say, the *j* ^th^ subunit) randomly, and rotate its orientation by a random angle *θ*_*j*_ between ±1.5 radian. This move may alter the inter-protofilament distances of many of subunits. We recalculate inter-protofilament distances and the corresponding total energy of the new micro-state as ${ℰ}_{\text{new}}$. The move is accepted with appropriate weight $\exp\left(\beta ({ℰ}_{\text{new}}-\;{ℰ}_{\text{in}})\right)$ according to the Metropolis algorithm [39, 40]. We repeat this procedure for large number of times (≈ 10^10^MC steps) such that various measurable quantities have a steady state distribution. The acceptance probability based on this Metropolis algorithm in our simulations is between 1%−10%, depending on the combination of $E_{m}^{s}$ and $E_{m}^{b}$. Even though we go through a large number of Monte Carlo steps, in practice, this method may not achieve appropriate sampling of all states. To ensure proper sampling, we did the simulations independently using the umbrella-sampling method [41]. The “weights” for umbrella sampling were calculated using a modified energy-paving method described in [42] (see S2 Text). By comparing the outcomes, we have ensured that both the Metropolis and umbrella-sampling methods give the same results.

We choose the *x* distance of the tip, defined as *R*_*x*_ = *L*−*x*_*n*_, as an appropriate “order parameter” that can quantify the shape (or conformational “state”) of the semiflexible protofilament, where *x*_*n*_ is the *x* coordinate of the *N* ^th^ subunit and *L* = *Nb* is the total length of the protofilament (see Fig. 1(B)). Note that the conformational state of the semiflexible protofilament is related to the stability of the microtubule. For instance, when *R*_*x*_ ≈ 0 the protofilament is fairly straight with most of the lateral bonds intact—equivalent to the tubular state; we call this the “stable” state or “tubular” state of the microtubule. On the other hand, if the lateral interactions are broken, the protofilament goes to a peeled-off (curved) state with *R*_*x*_ → *L*; we call this the “unstable” state of the microtubule (see S3 Text and S1 Fig. for more details). From the Monte Carlo simulations, we compute *R*_*x*_ for each realization and obtain the probability distribution of *R*_*x*_, denoted as *P*(*R*_*x*_). This gives us the free energy landscape *F*(*R*_*x*_) = −*k*_B_T ln *P*(*R*_*x*_) that the tip of MT protofilament experiences as it unzippers. We then study the dynamics of the tip moving through this free energy landscape using Langevin dynamics simulations (see text below, and also S4 Text). Dissociation of subunits is also incorporated into the Langevin simulations by considering it as a non-equilibrium switching from one free energy landscape to another, with similar shape but of shorter length. From these simulations we compute the unzippering and the shrinkage velocities of the microtubule explicitly.

The parameters in our simulations are taken as close to the known MT parameters as possible (see Table 1). For example, subunit dimensions *b* = 8nm and *l* ^*o*^ = 6.5nm are the tubulin dimer length and diameter, respectively [17, 20]. Throughout this paper, we choose *l*_max_ = 1.2*l* ^*o*^, *θ* ^*D*^ = 0.4 rad and *θ* ^*T*^ = 0 [17, 20]. We perform a detailed parametric study by varying *k* ^*b*^ and *k* ^*s*^. By varying *k* ^*b*^ and *k* ^*s*^ (keeping everything else constant), we are essentially varying $E_{m}^{b}=k^{b}(1-\cos(\theta^{D}))$, $E_{m}^{s}=\frac{1}{2}k^{s}{\left(l_{\text{max}}-l_{i}^{o}\right)}^{2}$. $\Delta E=E_{m}^{s}-E_{m}^{b}$ is a parameter that captures the power struggle (competition) between the bending interactions and the lateral interactions, and it influences the MT stability. The results presented in the main text are for $E_{m}^{s}=8k_{\text{B}}T$, unless specified otherwise; the results for other parameters are given in Supporting Information (SI). First, we will discuss the results from the one-PF model, assuming no GTP cap. Then, we will discuss the roles of the GTP cap and multiple PFs, specifically.

**Table 1:** Parameters used in the paper.

*b*	Length of each subunit (= 8 nm)
$E_{m}^{b}$	Bending strain energy per subunit stored inside the naturally curved GDP microtubule lattice when it is constrained to be straight(= *k* ^*b*^(1−cos(*θ* ^*D*^)))
$E_{m}^{s}$	Maximum strength of the lateral interaction ($=\frac{1}{2}k^{s}{\left(l_{\text{max}}-l^{o}\right)}^{2}$)
*k* ^*b*^	Bending stiffness of the PF
*k* ^*s*^	Stiffness constant of the lateral interaction
*L*	Total length of a protofilament(= *Nb*)
*L*_*c*_	Length of the peeled-off segment of a protofilament
*l*_*i*_	Distance between *i* ^*th*^ subunit of the protofilament with its fixed lateral neighbor
*l*_*i*,*α*_	Pairwise distance between the node of *i* ^*th*^ subunits of *α* ^*th*^ and *α*+1^*th*^ protofilament in multi-protofilament model
*l*_*max*_	Maximum length of lateral interaction beyond which the lateral spring bond is “broken”
*l* ^*o*^	Equilibrium length of lateral interaction
*N*	Number of subunits in a protofilament
*N*_*c*_	Number of subunits in the peeled-off segment of a protofilament
*r*_*c*_	The radius of circular arc made by peeled-off segment of a protofilament(= *b*/*θ* ^*D*^)
*R*_*x*_	*x* distance of the tip (*R*_*x*_ = *L*−*x*_*n*_, where *x*_*n*_ is the *x*-coordinate of *N* ^*th*^ subunit)
*α*	Protofilament index in the Multi-protofilament model
Δ*E*	$=E_{m}^{s}-E_{m}^{b}$, is the power-struggle parameter
*θ*_*i*_	Relative angle between longitudinally adjacent subunits *i* and *i*+1
$\theta_{i}^{o}$	Preferred intrinsic angle between longitudinally adjacent subunits
*θ* ^*D*^	Preferred intrinsic angle between longitudinally adjacent D subunits (= 0.4 rad)
*θ* ^*T*^	Preferred intrinsic angle between longitudinally adjacent T subunits (= 0 rad)

## Results

### Thermal fluctuations are crucial when $E_{m}^{b}\approx E_{m}^{s}$

**Figure 2:**
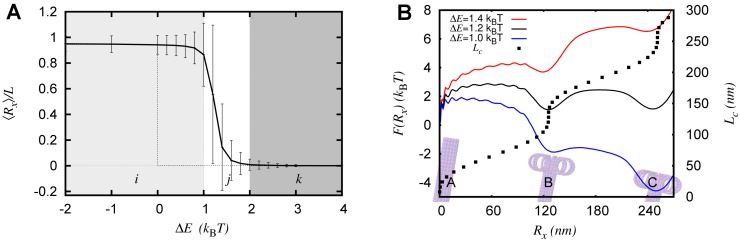
Role of thermal fluctuations. (A) ⟨*R*_*x*_⟩ vs Δ*E* with (solid), and without (dotted), thermal fluctuations. Shaded regions correspond to peeled off (i), bounded (k), and transition/mixed (j) states of microtubule. The vertical bars represent standard deviation, and imply coexistence of diverse set of conformations in *j*. (B) The free energy *F*(*R*_*x*_) when Δ*E* = 1.0, 1.2, 1.4*k*_B_ *T* (bottom to top, continuous curves). *R*_*x*_ vs *L*_*c*_ relation (Eq. 7) is plotted along the right hand side ordinate (*Y*_2_ axis). Microtubule pictures are embedded in the background to indicate that at *R*_*x*_ = 0 we have a tubular MT; at *R*_*x*_ ≈ 120nm we have a partially peeled off MT with the unzippered PF forming a circle-like conformation; at *R*_*x*_ ≈ 240nm we have a partially peeled off MT with the unzippered PF forming a conformation close to two full circles. Each of these states correspond to local minima in the free energy. These results are obtained by employing umbrella sampling method.

First, we investigate the stability of GDP MT within the single-PF model by measuring ⟨*R*_*x*_⟩ as a function of the power struggle parameter $\Delta E=E_{m}^{s}-E_{m}^{b}$ (see Fig. 2(A)). The angular bracket, ⟨…⟩, denotes averaging over many conformations in the ensemble. Zero-temperature theories of MT mechanics, with no GTP cap, predict only two states: *R*_*x*_ = 0 (fully bound) when $E_{m}^{s}>E_{m}^{b}$ or *R*_*x*_ ≃ *L* (fully peeled off) when $E_{m}^{s}<E_{m}^{b}$ (Fig. 2(A), dotted curve) [23, 32, 33]. Our simulations show that the thermal fluctuations alter the ⟨*R*_*x*_⟩ curve significantly, when 0 < Δ*E* < 2*k*_B_ *T* (Fig. 2(A), continuous curve). Even when lateral energy is slightly higher than the bending energy (e.g., Δ*E* = 1 *k*_B_ *T*), it is found that the PF is completely peeled off. This can be understood by noting that the entropy of the peeled-off state is higher, and thermal fluctuations tilt the scales in favor of unzippering. This shows that, unlike the prevalent notion gathered from zero-temperature theories, MT can be unstable even when the lateral interaction strength is higher than the bending interaction strength. Additionally, in the transition zone (Fig. 2(A), region *j*) the standard deviation (vertical bars) in *R*_*x*_ is comparable to ⟨*R*_*x*_⟩, implying the presence of a wide spectrum of states—from completely bound to completely peeled off. In SI, we show that the results are similar for a wide range of parameters (see S2 Fig. in SI). We also show that the results are similar even if one uses different functional forms for the potential energy used in the Model section (see S3 Fig. and S1 Text in SI).

### Free energy has multiple minima, implying the existence of diverse partially peeled-off states

To gain insights into the shrinkage and unzippering of GDP MT, it is important to understand the free energy landscape through which the protofilaments unzipper. The relevant free energy is *F*(*R*_*x*_) = −*k*_B_ *T* ln *P*(*R*_*x*_), where *P*(*R*_*x*_) is the probability of finding the microtubule PF tip at a distance *R*_*x*_. When the bending interaction is much stronger than the lateral interaction (Δ*E* ≪ 0), as expected from Fig. 2(A), the GDP protofilament is fully peeled off; therefore, we expect a free energy that has one major minimum near the completely peeled-off state, given by *R*_*x*_ ≈ *L*−*r*_*c*_ sin (*L*/*r*_*c*_) (see S3 Text for details). In the other extreme, when Δ*E* ≫ 0, the MT is highly stable (tubular state) and one expects a major minimum in the free energy near *R*_*x*_ = 0 (see S4 Fig. in SI). Interesting physics is seen in the regime 2*k*_*B*_ *T* > Δ*E* > 1 *k*_*B*_ *T* (regime *j* in Fig. 2(A)); here we see three prominent minima in the free energy—one near *R*_*x*_ = 0, one near *R*_*x*_ = *L*, and another one in the middle, near *R*_*x*_ = 2*πr*_*c*_ (see continuous curves in Fig. 2(B)). This is the regime where ⟨*R*_*x*_⟩ shows a transition from a tubular to a fully peeled off state. This indicates that, for these parameter values, even an MT composed of only GDP tubulin (no GTP cap) can co-exist in multiple states—in tubular, partially peeled-off, and completely peeled-off states. Note that *r*_*c*_ = *b*/*θ* ^*D*^ is the length scale due to the intrinsic curvature of the GDP tubulin, and *R*_*x*_ = 2*πr*_*c*_ ≈ 125nm is the state where the peeled-off portion of the protofilament prefers to be in the conformation of a full circle of radius *r*_*c*_ ≈ 20nm.

These minima reveal interesting polymer physics behind the MT protofilament unzippering. The source of these minima is a three-way competition between bending energy, lateral interaction energy and entropy. If any of these terms are set to zero, the minima will disappear. However, the signature of these minima can be seen by plotting the zero-temperature relation (see S3 Text for details),

$$R_{x}=R_{x}^{0}=L_{c}-r_{c}\sin(L_{c}/r_{c}),$$ (7)

derived from simple geometrical arguments (see Fig. 2(B) dotted curve). When *R*_*x*_ ≈ 125nm, there are many *L*_*c*_ values (plotted along the right hand side ordinate, that is, *Y*_2_ axis) for which the bending energy of the peeled-off portion is zero, implying high “entropy” (hence, free energy minimum) here. We can have a better understanding of these minima as in the following manner. By definition, $P(R_{x})=\sum_{N_{c}=1}^{N}\exp(-\Delta EN_{c})𝓟(R_{x},N_{c})$, where $𝓟(R_{x},N_{c})$ is the distribution function of an intrinsically curved semiflexible polymer of length *L*_*c*_ = *N*_*c*_ *b* with no lateral interactions (see S5 Text for details). Given the high bending stiffness of protofilaments, $𝓟(R_{x},N_{c})$ will be a highly peaked function around the zero-temperature value of $R_{x}=R_{x}^{0}$. If we assume $𝓟(R_{x},N_{c})$ as a Gaussian peaked at $R_{x}^{0}$, we see that *P*(*R*_*x*_) has multiple peaks and the corresponding free energy has multiple minima (see S5 Text for details).

### Unzippering rate calculation and prediction of protofilament interaction parameters

**Figure 3:**
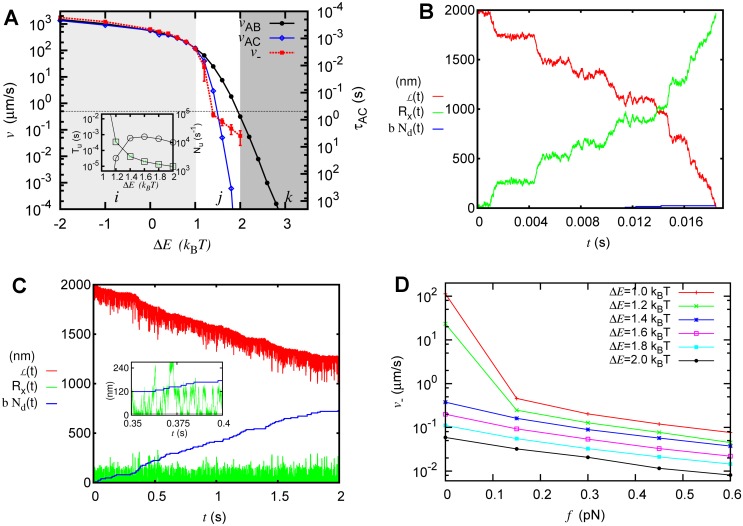
Unzippering and shrinkage of MT. (A) Unzippering velocities *v*_*AB*_, *v*_*AC*_, and effective shrinkage velocity (*v*_−_) compared to experimentally measured MT shrinkage velocity (horizontal line). The timescale corresponding to the blue points, *τ*_*AC*_ = 250nm/*v*_*AC*_ is shown along the right hand side ordinate (*Y*_2_ axis). The vertical bars represent standard error. (Inset): Average lifetime of unzippered PFs (*T*_*u*_, squares) and the number of unzippering events per second (*N*_*u*_, circles). (B) and (C): Examples of typical time-trajectories from the Langevin simulation for Δ*E* = 1.2*k*_B_ *T* and Δ*E* = 1.4*k*_B_ *T*, respectively; here *R*_*x*_(*t*) is the tip position of the unzippered PF (green), *bN*_*d*_(*t*) is the length change due to dissociation of the subunits (blue), and ℒ(*t*) is the observable MT length (red). (B) Unzippering of the protofilament (see *R*_*x*_(*t*)) is the dominant mechanism in the change in observed length when $\Delta E\underset{\approx }{<}1.2k_{\text{B}}T$. (C) The dissociation of subunits becomes the dominant mechanism of length change in MT when $\Delta E\underset{\approx }{>}1.4k_{\text{B}}T$. Here, one can see that unzippering of protofilament leads to partially peeled-off states where one can simultaneously observe subunit dissociation and existence of ram’s horns—see inset in (c), where we have zoomed into a small time window of the main figure. (D) Effect of force (per protofilament) on the shrinkage velocity of MT.

How much time does an MT protofilament take to peel off and reach a given value of *R*_*x*_? How much of the protofilament will peel off, before the timescale of depolymerization? These are important questions to address because, as we argued earlier, the timescale of peeling off should not be very different from the experimentally known shortening timescale of GDP microtubules. A simple way to compute this timescale of unzippering is to calculate the time it takes to move from one location to another in the free energy landscape. This can be achieved using a well-known calculation in physics known as the “first passage time” calculation [2, 43]. This is also common in polymer physics where the N-degree of freedom problem is converted to an essential 1-degree of freedom question [44, 45]. Extending this method to our problem, from the free energy landscape, we compute *τ*_*AB*_ and *τ*_*AC*_, the mean first passage times to move from *R*_*x*_ = 0 (state A) to *R*_*x*_ = 125nm (state B), and to move from *R*_*x*_ = 0 to *R*_*x*_ = 250nm (state C), respectively. Knowing the free energy, the first passage time can be computed as

$$\tau_{AB}=\frac{1}{D}\int_{A}^{B}\exp\left[-\frac{F(R_{x})}{k_{\text{B}}T}\right]\left\{\int_{R_{x}}^{B}\exp\left[\frac{F(u)}{k_{\text{B}}T}\right]du\right\}dR_{x},$$ (8)

where *D* is the effective diffusion coefficient of the tip calculated using $D=\frac{k_{\text{B}}T}{6\pi \eta a}$, where *η* is the viscosity of water and *a* is the effective size of the peeling blob. This equation is the standard way to compute first passage time in an energy landscape, as described in refs. [2, 43]. Here, we consider *a* = 40 nm—the size of a circle made out of GDP MT—to set a conservative lower bound on the diffusion constant. Note that *τ*_*AB*_ is the rough timescale for the PF to partially peel off and form a ram’s horn of one circle of radius *r*_*c*_. Similar to *τ*_*AB*_, we also calculated *τ*_*AC*_, which is plotted in Fig. 3(A) as the right hand side ordinate (*Y*_2_ axis) of the blue curve (also see S4 Fig. in SI). Using these timescales, we compute the local peeling-off (unzippering) velocities as *v*_*AB*_ = 125nm/τ_*AB*_, *v*_*AC*_ = 250nm/τ_*AC*_. *v*_*AB*_ and *v*_*AC*_ are the mean velocities with which the MT protofilament will peel off from A to B and A to C, respectively and are plotted in Fig. 3(A); (see S4 Fig. in SI for the corresponding time scales *τ*_*AB*_ and *τ*_*AC*_). We have also obtained the unzippering dynamics using Langevin simulations of the tip through the free energy landscape (as described in S4 Text); a set of typical time trajectories from these simulations are shown in Fig. 3(B) and (C) (see green curves). When one compares *v*_*AC*_ with the experimentally observed [2, 26] MT shrinkage velocity of GDP microtubule ($v_{-}^{\text{exp}}\approx 0.5\mu \text{m/s}$, horizontal line Fig. 3(A)), one finds that only in the regime *j*, where Δ*E* ≈ *k*_*B*_ *T*, *v*_*AC*_ is comparable to $v_{-}^{\text{exp}}$. Interestingly, in the bending-dominated regime ($E_{m}^{s}\ll E_{m}^{b}$), the unzippering velocity is 1000 times faster than the experimentally seen shrinkage velocity (e.g., see Fig. 3(A), Δ*E* ≈ −2*k*_B_ *T*); this questions the notion in the literature that the strength of the microtubule protofilament curvature energy dominates over the lateral interaction energy [21, 23, 25, 32]. Since $v_{AC}\approx v_{-}^{\text{exp}}$ only when Δ*E* ≈ *k*_B_ *T*, our results suggests that experimentally observed microtubule shrinkage rate is achievable only when entropy competes with energy. One may note that when the free energy landscape is tilted with a bias towards the tubular state (for example, Fig. 2(B), Δ*E* = 1.4*k*_B_ *T*), the first passage time *τ*_*AB*_ and *τ*_*AC*_ are finite; however, the time it takes for a PF to completely peel off (as opposed to partially peel off) diverges (see S4 Fig. in SI). In other words, the protofilaments will fluctuate in partially peeled-off states, as seen in the Fig. 3(C) *R*_*x*_(*t*) trajectory (see details below). This has interesting significance in understanding the complete shrinkage dynamics, as discussed below.

### Shrinkage of MT: Interplay between unzippering and dissociation

The results obtained above suggest a new picture of MT shrinkage: The competition between lateral, bending and thermal energies results in a free energy landscape with multiple minima; the shape of the landscape prevents uncontrolled (fast) unzippering, and restricts the PFs to fluctuate between a set of partially peeled-off states. However, this also introduces the following new questions: What is the role of subunit dissociation during MT shrinkage? How does the interplay between dissociation and unzippering lead to the observed MT shrinkage?

To answer these questions, we perform simulations combining Langevin dynamics of unzippering with the stochastic dissociation of subunits [46, 47]. Note that, given the free energy, Langevin dynamics is the natural method to simulate a stochastic motion on the free energy landscape [40, 48] (also see Discussion section below). In the Langevin simulations here, we compute the tip position as a function of time (*R*_*x*_(*t*))—see Fig. 3(B) and (C) green curves; this enables us to calculate the observed length of the microtubule (ℒ) at any instant, as

$$ℒ(t)=ℒ(0)-R_{x}(t)l_{d}-bN_{d}(t),$$ (9)

where *l*_*d*_ is a geometric factor that relates *R*_*x*_ and ℒ, and *N*_*d*_ is the total number of subunits dissociated up to time *t*. ℒ(*t*) is plotted in Fig. 3 (B) and (C) (red curves). The details of the simulation is described in S4 Text. In these simulations, we assume that the MT subunits do not dissociate when the PF is in the tubular state (*R*_*x*_(*t*) near 0)—that is, when the lateral bonds are intact. From the Langevin simulations, we get the ℒ(*t*) (see S4 Text), and its slope gives us the average shrinkage velocity as

$$v_{-}=\frac{d\langle ℒ(t)\rangle }{dt}.$$ (10)

This velocity is plotted in Fig. 3(A) (red squares). As expected, for small or negative Δ*E* values, the shrinkage velocity is very high in comparison to the experimentally known shrinkage rate, and matches with the computed unzippering velocity. This reinforces our previous result that unzippering is the dominant mechanism of shrinkage for this range of parameters (regime *i*). For Δ*E* ≈ 1.4*k*_B_ *T*, the shrinkage velocity becomes comparable to the experimentally known shrinkage rate. As seen in Fig. 3(C) and Fig. 3(C) inset, our data predicts that *R*_*x*_ is distributed approximately between 0 to 240 nm with an average ≈ 50nm. We have also calculated the average lifetime of unzippered PFs (*T*_*u*_) and the number of unzippering events per second (*N*_*u*_). *T*_*u*_ is defined as the average time for which one would observe unzippered ram’s horns of length *R*_*x*_ > 10 nm. These are shown in Fig. 3(A) inset. When the free energy landscape is tilted towards the peeled-off state (small or negative Δ*E*), one would always observe ram’s horns (very large *T*_*u*_ and extremely small *N*_*u*_). When the free energy landscape is slightly tilted towards the tubular state, the ram’s horns appear and disappear frequently (also see Fig. 3 (C) inset). When combined, these findings demonstrate that when Δ*E* ≈ 1.4*k*_B_ *T*, the life-time of ram’s horns and number of unzippering events combine in such a way that the average shrinkage velocity is close to the experimental value. In other words, roughly in the middle of the *j* regime, we have the “right combination” of long enough life-times (*T*_*u*_) and frequent enough unzippering events (*N*_*u*_) to produce shrinkage with all experimentally known features.

From our simulations, the following picture emerges: when the landscape is tilted with a slight bias towards the tubular state (for example, Fig. 2(B), Δ*E* = 1.4*k*_B_ *T*), the PF will fluctuate in the landscape with a non-zero, but finite, average peeled-off length; this will not lead to any observable shrinkage (see Fig. 3(C) green curve). While the protofilaments are fluctuating in these partially peeled-off states (unzippering and re-zippering back), the dissociation will happen; this will alter the length of the protofilaments leading to observable shrinkage (also see S1 Video). Note that here, the unzippering and dissociation go hand in hand—that is, without thermally-driven unzippering, there will be no dissociation; with no dissociation, there will be no observable shrinkage in this regime. Therefore, the only physically feasible mechanism of shrinkage is in this parameter regime, where both unzippering and dissociation play crucial roles; our calculations reveal how these two different mechanisms work together during MT shrinkage.

Effect of force: There have been in vitro studies of how microtubule shrinkage gets affected by external pulling forces. For example, a recent paper by Akiyoshi et al. shows that when a pulling force is applied to an MT tip the shrinkage velocity decreases exponentially with the magnitude of the applied force [49]. Here we extend our model to incorporate the role of the external force, and examine how the pulling force will change the shrinkage velocity within this model. The simplest way to incorporate external force into our model is to assume that the pulling force tilts the free energy landscape, such that *F*(*R*_*x*_, *f*) = *F*(*R*_*x*_, 0) + *fR*_*x*_, where *F*(*R*_*x*_, *f*) is the new free energy landscape in the presence of pulling force *f* at the tip, on a given protofilament. Incorporating an external generalized force by tilting the landscape is often done in polymer physics [44] as well as in various biological problems, such as the motion of molecular motors under external force (see e.g. ref. [50])). In fact, in the context of microtubules, Armond and Turner have used a similar approach (that is, coarse-graining and tilting) to investigate the problem of force transduction due to Dam1 ring [51]. The shrinkage velocity in this landscape is computed by performing Langevin simulations as discussed above. The result is shown in Fig. 3(D), in which the shrinkage velocity decreases with force, displaying interesting features. When Δ*E* ≥ 1.4*k*_B_ *T*, the *v*_−_ decreases exponentially as seen in the experiment. However, as parameters approach the *i* regime, the system shows a different behavior for small and large forces. This can be understood if we note that in these parameter regimes, the nature of the shrinkage itself changes from unzippering-dominated to a combined mechanism comprising unzippering and dissociation.

### A single GTP cap can delay the peeling off beyond polymerization timescales

**Figure 4:**
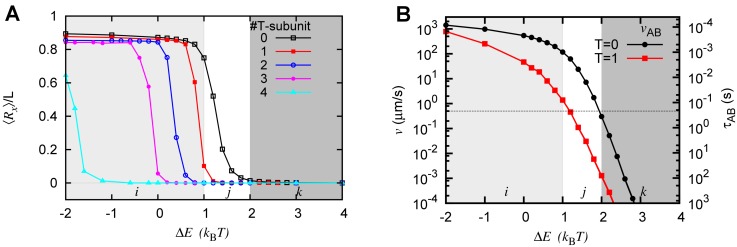
Effect of GTP-cap. (A) ⟨*R*_*x*_⟩ as a function of Δ*E* for different sizes of GTP cap. Here we simulate a single protofilament of *L* = 19*b* by fixing $E_{m}^{s}=8k_{\text{B}}T$ in the presence of different numbers of T-subunits (1, 2, 3 or 4) at the plus end of MT. (B) A single GTP cap (T = 1) decreases the initial unzippering velocity, when compared to zero cap (T = 0). The timescale corresponding to the red dots (squares) is in the *Y*_2_ axis. These results are obtained by employing the umbrella-sampling method.

So far, we discussed the statistical mechanics of microtubule protofilaments made of only GDP subunits. Here, we examine the effect of GTP cap on the stability and unzipping timescales of microtubules. We simulate the protofilament with *m* GTP subunits at the tip, while the rest of the bulk is made of (*n*−*m*) GDP subunits. As discussed in the Model section, GTP cap subunits have no intrinsic curvature (*θ* ^*T*^ = 0), and hence, prefer to be straight; this results in relatively stable filaments as reflected in the ⟨*R*_*x*_⟩ plotted in Fig. 4 (A)—the insertion of GTP subunits make ⟨*R*_*x*_⟩ essentially smaller when compared to the case with no cap (see S5 Fig. in SI). A single GTP cap shifts the stable-to-unstable transition by ≈ 0.5*k*_B_ *T*, and two GTP subunits make the microtubule fully stable (⟨*R*_*x*_⟩ = 0) for any parameter in the *j* regime. It is interesting to note that even in the presence of thermal fluctuations, our simple model suggests that one or two layers of GTP cap will make the system stable, if the parameters are in the *j* regime. This is also compatible with experimental observations in refs. [52, 53]. We have presented more results in S5 Fig., where we have simulated protofilaments with *m* GTP-subunits at the tip, keeping the GDP bulk constant at *n* = 19 subunits, with the total length of the protofilament being *m*+*n*.

In the presence of cap, we also computed the free energy landscape, the unzippering timescales and velocities, in a similar manner as discussed earlier. The cap at the tip creates a large barrier at *R*_*x*_ = 0, trapping the filament in the straight conformation (See SI S5 Fig.). This delays the unzippering process and increases the time taken to reach from A to B (*τ*_*AB*_), for example, by a factor of 100 for one GTP subunit (see Fig. 4(B) right hand side ordinate (*Y*_2_ axis)). The corresponding unzippering velocity (*v*_*AB*_) decreases equivalently. Interestingly, when Δ*E* ≈ *k*_B_ *T*, *τ*_*AB*_ is much larger than the typical polymerization time (≈ 10^−2^s per subunit for ≈ 20*μ*M) [2]. This implies that, in this *j* regime, the polymerization is very likely to happen much before any unzippering taking place, with just one cap. When *E*_*b*_ ≫ *E*_*s*_, single cap has little effect and the filament will peel off quickly. This is also consistent with the proposal of Dimitrov et al. that the rescue can start from GTP remnants in a microtubule [54].

### Multiprotofilament model suggests cooperative unzippering of protofilaments while retaining the main features of the single protofilament model

**Figure 5:**
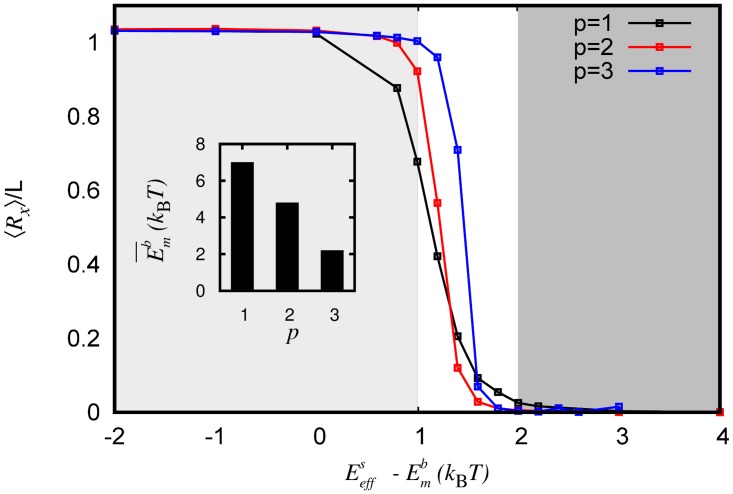
Multi-protofilament simulations. Multi-protofilament model results are comparable to the single filament model if one scales lateral interaction energy appropriately. Average x position of the tip ⟨*R*_*x*_⟩ of a GDP protofilament as a function of the effective power-struggle parameter $E_{\text{eff}}^{s}-E_{m}^{b}=\Delta E$, in the multi-protofilament version of the model. These simulations are performed for *L* = 15*b* in a 3-protofilament system. The black curve (*p* = 1) represents the case where only one protofilament out of the three is bendable (i.e., $E_{\text{eff}}^{s}=8k_{\text{B}}T$). The red curve (*p* = 2) represents the case where two out of three protofilaments are allowed to bend, and the remaining one is stationary (i.e., $E_{\text{eff}}^{s}=6k_{\text{B}}T$). The blue curve (*p* = 3) represents the case where all three filaments are bendable (i.e., $E_{\text{eff}}^{s}=4k_{\text{B}}T$). The inset shows the minimum strength of bending interaction ${\bar{E}}_{m}^{b}$ required to destabilize a microtubule (i.e. make ⟨*R*_*x*_⟩ ≥ *L*/2) for different cases of *p*. It suggests that as more and more protofilaments get stabilized (*p* decreases), one needs stronger curvature energies to destabilize a microtubule. These results are obtained by using the Metropolis Monte Carlo method.

We have also simulated a microtubule with three protofilaments. This mimics reality, in which each PF has two neighbors, all are bendable, and interacts laterally with both the neighbors. Note that the one-bendable-PF model is a special case of the current three-PF model, when two of the PFs are fixed. In the one-PF model, the effective lateral interaction energy per bendable protofilament $E_{\text{eff}}^{\text{s}}=(4+4)k_{\text{B}}T=8k_{\text{B}}T=E_{m}^{s}$, as is used in the one protofilament model earlier (*p* = 1); here, 4*k*_B_ *T* is the interaction energy per lateral spring bond. When only two PFs are bendable (*p* = 2), $E_{\text{eff}}^{\text{s}}=(4+4+4)k_{\text{B}}T/2=6k_{\text{B}}T$. When all the three PFs are bendable(*p* = 3), $E_{\text{eff}}^{\text{s}}=(4+4+4)k_{\text{B}}T/3=4k_{\text{B}}T$. In Fig. 5, we plot ⟨*R*_*x*_⟩ of one of the PFs as a function of $E_{\text{eff}}^{\text{s}}-E_{m}^{b}$ for one, two, and three bendable PFs. All the curves are nearly similar, suggesting that the one-PF model, with lateral energy interpreted appropriately, is adequate to gain insights into the more complicated multi-protofilament problem. In other words, our results indicate that whenever the bending interaction is ≈ 1 to 2*k*_B_ *T* less than the effective spring interaction each protofilament “experiences,” the MT shows a transition from the tubular state to the peeled-off state. The free energy curves for equivalent parameters are also similar (see S6 Fig. in SI), suggesting that our results are robust even within a multi-PF model.

Using the three variants we discussed, we can also address how stabilization of a individual protofilament affects the stability of the microtubule. Specifically, we ask the following question: What is the minimum bending energy per bendable protofilament (${\bar{E}}_{m}^{b}$) required to make the microtubule unstable (that is ⟨*R*_*x*_⟩ > *L*/2), and how does this change when we disallow a neighbor to bend (or fluctuate)? The result is shown in the inset of Fig. 5. For case three (*p* = 3), the destabilizing bending energy ${\bar{E}}_{m}^{b}\;\approx \;2k_{\text{B}}T$. For *p* = 2 case, when one particular protofilament is not allowed to bend, ${\bar{E}}_{m}^{b}\approx 4.5k_{\text{B}}T$. In other words, stabilizing one protofilament makes the microtubule more stable. For *p* = 3 case, when two of the thee protofilaments are stabilized, ${\bar{E}}_{m}^{b}$ further increases to ≈ 7*k*_B_ *T*. This demonstrates that for a given set of parameters, ⟨*R*_*x*_⟩ decreases (MT becomes stable) as one fixes (stabilizes) one or more protofilaments; this is a clear reflection of the fact the effective lateral interaction energy per protofilament increases. Equivalently, it also implies that the more the number of fluctuating protofilaments, the more concerted the MT catastrophe is. This finding may have ramifications in actual microtubules, where the presence of GTP caps in neighboring protofilaments may affect the stability of a given protofilament. Our finding is also consistent with the experimental observation of Hendricks et al., in which they found that a dynein tethered to a few protofilaments can lead to an enhanced stability of the whole microtubule [55]. The finding may be also important in vivo, where there are also various factors that stabilize or destabilize microtubule protofilaments and regulate their dynamics.

## Discussion

Our results suggest a few major points: (i) The right parameter regime for bending and lateral interactions of microtubule protofilaments has to be the regime where Δ*E* ≈ *k*_B_ *T*, and in this regime, thermal fluctuations are crucial for microtubule stability. (ii) The notion that the dominance of bending energy, in the GDP tubulin, is what drives the unzippering of microtubules [21–23, 25, 32] is paradoxical as it would not produce experimentally observed shrinkage velocity. (iii) Our findings can provide a *consistent description* by which one can reconcile the two main features observed during shrinkage, namely the peeling off and depolymerization. Therefore, our findings have important implications in the shrinkage dynamics of microtubules and may provide interesting insights into the mechanism of MT regulation.

**Bending interaction cannot dominate lateral interaction:** All the results we obtained in this paper suggest one point: bending energy cannot dominate over lateral interaction energy ($E_{m}^{b}$ cannot be larger than $E_{m}^{s}$). For example, when $E_{m}^{s}-E_{m}^{b}=-2k_{\text{B}}T$, the free energy landscape is highly tilted toward the peeled-off state, and the unzippering velocity of GDP microtubule is 1000 times larger than the experimentally reported shrinkage velocity (Fig. 3(A)). The study with GTP cap (Fig. 4) suggests that when Δ*E* = −2*k*_B_ *T*, even multiple layers of GTP cap cannot stabilize the microtubule; however, existing experiments suggest that one or two layers of cap can stabilize microtubules, implying that Δ*E* cannot be highly negative. Our study of multiprotofilaments is also consistent with these findings and indicate that Δ*E* has to be ≈ *k*_B_ *T*. Our results are in contrast with the existing notion in many of the theoretical papers [21–23, 25, 32], where the parameter Δ*E* is considered to be highly negative. Since these earlier works did not account for thermal fluctuations, a negative Δ*E* was necessary in their model to produce unzippering. However, it may be noted that the parameters estimated in refs [31, 56] are consistent with our findings and fall in the regime *j*.

**Figure 6:**
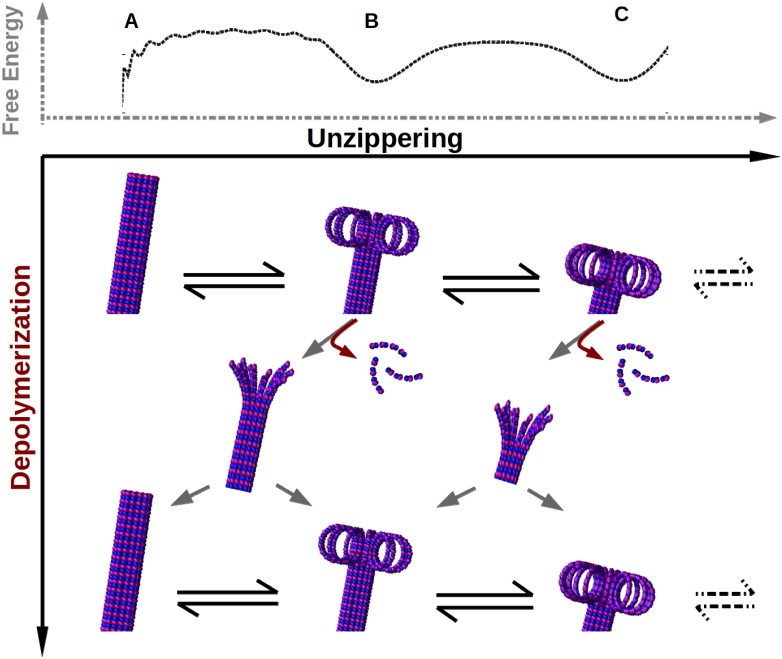
Insight into the mechanism of MT shrinkage. The interplay of bending interactions, lateral interactions and thermal fluctuations give rise to a complex free energy landscape (dotted black curve; also see Fig. 2) in which GDP-protofilaments fluctuate between several partially peeled-off states. These partially peeled-off states would manifest as ram’s horn-like structures. Subunits from MT can dissociate from the protofilaments while they fluctuate in these states. This is a description that can combine both the mechanisms of shrinkage, unzippering and depolymerization, with timescales that are consistent with experiments.

**A new model for the shrinkage mechanism:** From our findings, a new description for the mechanism of microtubule shrinkage emerges; the details are as follows: As we find in the results, the lateral interaction energy competes with the bending energy and thermal energy to determine the shape of the free energy landscape. The tilts, and the local minima in the landscape prevent uncontrolled (fast) unzippering of microtubule protofilaments. Nonetheless, thermal fluctuations ensure a finite amount of unzippering, which is fast initially (*v*_*AB*_) when compared to the experimentally known shrinkage rates. Note that it is a novel feature of our results that thermal fluctuations are important for the unzippering itself. This controlled unzippering results in protofilaments fluctuating in a set of partially peeled-off states—a biased Brownian walk on the free energy landscape. The prediction of highly probable partially peeled-off states in our model is consistent with the observation of the ram’s horns-like structure in various experiments [19, 20]. The nature of the free energy landscape ensures that the timescale for the peeled off protofilaments to go beyond the state *B* is comparable to the known estimates of depolymerization timescales [2]. Therefore, as seen from the Langevin dynamics simulations, the PFs fluctuating in the partially peeled-off states undergo depolymerization and reduce their length. It is also a new feature of our model that we provide insights into the emergence of timescales that are relevant for unzippering and shrinkage. Thus, we have a plausible novel mechanism by which one can reconcile the two known pictures of microtubule shrinking—unzippering and depolymerization (see Fig. 6, also see S1 Video in SI). Even though some earlier works discuss unzippering followed by dissociation, they do not provide a microscopic mechanism to explain the unzippering that is consistent with all known aspects [31, 56, 57]. In those works, unzippering is a kinetic event with a given rate, the origin of which is either unknown or driven by the curvature energy of the GDP tubulin. On the other hand, we have an alternative picture where the unzippering rate emerges from the microscopic interactions, with thermal fluctuations playing a crucial role. In summary, the novelty here is that, unlike earlier works, our work provides a consistent picture with all of the following aspects: (a) thermal fluctuations that are inherent in experiments, (b) a physical mechanism for peeling off (and re-zippering back) with sensible timescales, (c) an accounting for sufficiently long ram’s horns that are observable in experiments, and (d) obtaining the experimentally seen shrinkage velocity, such that all of the above are satisfied.

**Regulation of microtubule dynamics:** As we see in our results, *j* regime is the regime where the microtubule shows a transition from a peeled-off state to a tubular state. This implies that a small change in Δ*E* (≈ *k*_B_ *T*) per subunit can alter the stability of the microtubule. Many of the small molecules/proteins may make use of this feature to regulate microtubule stability and dynamics. Indeed the literature is replete with examples of small molecules/proteins that can regulate MT stability and dynamics, even if present in very small concentrations [6, 58–60]. On the other hand, in the *i* regime, even a significant change in Δ*E* may not make any significant change in the MT stability, suggesting that the microtubule regulation is easier if the parameters fall in the *j* regime.

Our results also suggest that the cap can delay the catastrophe for a long time—for a period longer than the typically known polymerization timescales. This suggests that if there are GTP islands in the middle of a microtubule, the catastrophe will be arrested for a duration long enough for rescue. This is consistent with the known experimental findings of Dimitrov et al., in which the rescue starts from GTP remnants in a microtubule [54]. Even in the absence of a cap, the temporary arrest at the minimum at *B*, combined with depolymerization and thermally-driven re-zippering could also, in principle, take the filament back to *A* and, thus, trigger the possibility of a rescue. However, this will depend on the probability of finding a neighbor, when the filament fluctuate backs to *A*, and the probability of polymerization before the filament fluctuate backs to *B*. Nevertheless, a clear understanding of rescue would require a detailed study that takes into account the fluctuations of the peeled-off protofilaments, in the presence of other protofilaments, and is beyond the scope of the current paper.

**Suggestion for new experiments to test our predictions:** In this computational study we have obtained appropriate parameters for microtubules to ensure that the shrinkage velocity computed is comparable with the experimentally observed value. Our study also explains, in a consistent manner, the experimentally observed ram’s horns in tandem with the depolymerization of subunits leading to shrinkage of microtubules. Our prediction that a single layer of cap can stabilize microtubules, as mentioned earlier, is also consistent with the known experiments so far. Our simulations also obtain exponentially decreasing shrinkage velocity with external force, as seen in experiments. Apart from all these, our study provides many new interesting predictions that are testable in appropriately designed experiments.

The complete protofilament dynamics that we predict is, in principle, testable if one can have measurement techniques with appropriate resolution in space and time. This may not be possible currently; however, we suggest a way to measure the average value of *R*_*x*_ by performing experiments to study shrinkage of microtubules by diluting the free tubulin concentration to zero (dilution assay). After dilution, the microtubules will shrink with the typical shrinkage velocity; during this shrinkage, one can freeze the system at various time intervals and obtain *R*_*x*_ using electron micrograph imaging (a procedure similar to ref [61]). Doing this for a population of microtubule protofilaments selected without any bias is predicted to yield a distribution of *R*_*x*_ in the range of 0 to 200nm with an average of ≈ 50nm (see Fig. 3 (C) green curve). Through this experiment, one may also test our prediction that there is a non-zero probability of observing individual protofilaments with a zero peeled off length.

**Equivalent approaches and alternative methods**: In this paper we have discussed one way of addressing the problem of microtubule shrinkage. The problem can also be approached using other equivalent methods. As we have mentioned in the earlier sections and in SI, there are many equivalent ways of performing a similar simulation. For example, one may use different functional forms for potential energies, as discussed in SI S1 Text. Once the free energy function is computed, one may also choose to use other methods such as writing down and solving the corresponding Focker-Planck equations systematically. All these methods are equivalent and are expected to give similar results.

A method alternative to ours is to perform a coarse-grained Brownian dynamics simulation of protofilaments, accounting for all subunits with bending and lateral interactions. Equivalently, one may also perform molecular dynamics simulations with more microscopic details added into the model. However, given that typical experimentally-observable MT shrinkage timescales are of the order of seconds (or higher), it will be nearly impossible to access such large timescales in these simulations using alternative methods such as Brownian/Molecular dynamics of protofilaments. One may also use an alternative model; unlike the allosteric model for tubulin conformation (i.e., GTP/GDP-dependent curvature) we used, one may opt for the “lattice model” as proposed in some of the works, where both GTP-bound and GDP-bound tubulin subunits have curved conformation in solution. However, when GTP-bound subunit gets assimilated into a microtubule, it forms lateral interactions of higher strength, which provide necessary energy to straighten GTP-subunits and, consequently, assemble the tubular supra-structure [30, 59, 62, 63]. We believe that incorporating the lattice-model will not alter our GTP-cap results, since the stronger lateral interactions can compensate for the probable curvature of the cap, providing a similar effect.

**Limitations of our work:** One of the limitations of our model is that we do not consider polymerization kinetics of subunits. However, given that our focus is on understanding shrinkage dynamics, the findings can be tested in specially designed experiments (for example, dilution experiments where polymerization is absent), as discussed above. Another limitation is that we do not explicitly account for all of the 13 protofilaments in our model. However, we have examined the multiprotofilament effects using a minimal 3-protofilament model and found the results to be in line with the single protofilament model. Due to geometric effects, the coupling between curving of protofilaments and stretching of lateral bonds can depend on the number of protofilaments (radius of the microtubule cylinder) in the model. However, we have done our calculations for a range of effective parameters and have found that our conclusions are robust (see S6 Text and S7 Fig.). Therefore, we do not expect the conclusions to be significantly different even if one considers the complete microtubule with thirteen protofilaments.

**Summary:** In conclusion, we have studied the stability of microtubule filaments using a statistical mechanical model that takes into account bending of protofilaments, inter-protofilament interactions and thermal fluctuations. Using Monte Carlo simulations we reconstructed the free energy landscape of microtubule protofilaments and computed their unzippering dynamics through this landscape. We found that when the bending energy dominates over lateral interaction energy, the protofilaments unzipper very fast—with a timescale that is much smaller than the experimentally observed shrinkage timescale. To obtain experimentally compatible unzippering and shrinkage, we find that the bending energy of a PF has to be comparable to the lateral interaction energy (Δ*E* ≈ *k*_B_ *T*). This leads to the prediction that thermal fluctuations have a crucial role in unzippering itself. Using Langevin simulations, we combined unzippering dynamics and dissociation of subunits and computed the shrinkage dynamics of MT. This leads to a novel picture of MT shrinkage in which thermal fluctuations drive unzippering and the fluctuating protofilaments depolymerize to alter the length. In this picture, the unzippering rate naturally emerges from the interactions and the existence of ram’s horns of average length of the order of ≈ 50nm is predicted. All this is done in a consistent manner, such that the overall shrinkage velocity matches with the experimentally known shrinkage rate.

## Supporting Information

S1 Text Comment on functional forms of lateral and bending interactions. In this text we discuss other possible functional forms to describe lateral and bending interactions, and their effect on our results.
(PDF)

S2 Text Monte-Carlo simulations using umbrella sampling. This text describes the methodology for umbrella sampling simulations.
(PDF)

S3 Text Geometric relationship between *R*_*x*_ and *L*_*c*_. (PDF)

S4 Text Shrinkage velocity calculation using Langevin dynamics simulations. This text describes the methodology of Langevin dynamics simulation, to incorporate non-equilibrium dissociation of tubulin subunit, to calculate shrinkage velocity.
(PDF)

S5 Text Analytical understanding of multiple minima in the free energy. In this text we derive a simple theory to gain some analytic understanding of occurrence of multiple minima in the free energy.
(PDF)

S6 Text Coupling between bending and stretching of lateral bonds. In this text we discuss the effect of geometric coupling between the bending of protofilament and stretching of lateral interactions in multi-protofilament model and its effect on our results.
(PDF)

S1 Fig Supporting figures complementing SI text. The figure show geometric relation between *R*_*x*_ and *L*_*c*_, result of theoretical analysis and an example of extended free energy landscape used in Langevin dynamics simulations.
(PDF)

S2 Fig The results shown in Fig. 2 are similar for a wide range of parameters. (PDF)

S3 Fig Alternative energy functions for lateral interactions. (PDF)

S4 Fig *P*(*R*_*x*_), *F*(*R*_*x*_), and unzippering timescales. (PDF)

S5 Fig The influence of GTP-cap on the stability of microtubules. (PDF)

S6 Fig Free energy from the multi-protofilament model. (PDF)

S7 Fig The figure show that the results in the paper are robust for a wide range of *l*_*max*_ and *k* ^*s*^ combination. (PDF)

S1 Video Video of the shrinkage dynamics. Using the data from the Langevin dynamics simulations (main text; Also S4 Text in SI), we produced an animation movie of the shrinkage process of a microtubule protofilament. When Δ*E* = 1.2*k*_B_ *T* (top realization in the movie), the shrinkage is dominated by unzippering and the protofilament shrinks with a velocity much greater than the experimentally observed microtubule shrinkage velocity. When Δ*E* = 1.4*k*_B_ *T* (middle realization), the protofilament fluctuates in partially unzippered states and stochastic dissociation decreases its length. The shrinkage velocity in this case matches with the experimentally known shrinkage velocity. The bottom-most graphics shows the initial conformation of the protofilament.
To make the movie, we calculated the observed length ℒ(*t*) = ℒ(0)−*R*_*x*_(*t*)*l*_*d*_−*bN*_*d*_(*t*), where *R*_*x*_(*t*) is the tip position and *N*_*d*_ is the number of dissociated subunit as described in the main text. To make this video, we assumed that the peeled-off part of the protofilament conformations are equivalent to their zero temperature conformations.
(MOV)
