# Supplementary material for: Statistical Mechanics Provides Novel Insights into Microtubule Stability and Mechanism of Shrinkage
Source: PLoS Comput Biol. 2015 Feb 18;11(2):e1004099. doi: 10.1371/journal.pcbi.1004099 (PMC4333834; doi:10.1371/journal.pcbi.1004099)
Supplement: S1 Text — In this text we discuss other possible functional forms to describe lateral and bending interactions, and their effect on our results. (PDF) [file pcbi.1004099.s001.pdf]

Text S1. Comment on functional forms of lateral and bending interactions:

In the main manuscript, we defined lateral interactions using a breakable Hookean spring (see Eqs. 1 and 2 in the main text) and bending interactions using a worm-like chain model with a given intrinsic curvature (see Eq. 3 in the main text). Alternatively, one may also define equivalent other energy functions to describe the same physical phenomenon as done in refs. [1, 2]. Lateral interactions can be defined using Lennard-Jones potential as

$$E_i^{s,LJ} = E_m^s \left(\left(\frac{l^o}{l_i} \right)^{12} - 2 \left(\frac{l^o}{l_i} \right)^{6} \right), \qquad \text{(SEq. 1)}$$

where E_m^s is the maximum strength of lateral interactions and l^o is the equilibrium length. Or alternatively, using Morse potential as

$$E_i^{s,M} = E_m^s \left(1 - e^{-\delta(l_i - l^o)^2} \right), \qquad \text{(SEq. 2)}$$

where E_m^s and l_o are the maximum strength and equilibrium length of lateral interactions respectively. We have also performed simulations (for our single protofilament model) using these energy functions. The results are given in Fig. S3. Using these functional forms for energies does not alter the main findings of the paper.

Similarly, to describe the bending energy one can use a quadratic functional like $\frac{\kappa}{2}(\theta_i - \theta_i^o)^2$, where κ is the bending stiffness (similar to k^b) (for example see ref. [2] below). One can immediately see that if the deviation in θ_i is small, both worm-like chain form and quadratic form will give the same value $((1 - \cos(\theta_i - \theta_i^o)) \approx (\theta_i - \theta_i^o)^2/2)$. Since the protofilaments have reasonably high bending stiffness, the θ_i is unlikely to fluctuate too much and the $(\theta_i - \theta_i^o)$ will be indeed small. In fact, the typical fluctuation in θ_i will be $\frac{k_{\mathrm{B}}T}{\kappa}$. For the bending stiffness values used in the paper (in the range is $25k_{\mathrm{B}}T - 130k_{\mathrm{B}}T$) we find that the fluctuation in θ is indeed very small (0.04 radian to 0.008 radian).

References

[1] V Hunyadi and Imre M. Jánosi. *Metastability of microtubules induced by competing internal forces.* Biophysical Journal **92** (May), 3092–3097 (2007).

[2] David Sept and Fred C Mackintosh. *Microtubule Elasticity : Connecting All-Atom Simulations with Continuum Mechanics.* Physical Review Letters **018101** (January), 1–4 (2010).
