# Supplementary material for: Statistical Mechanics Provides Novel Insights into Microtubule Stability and Mechanism of Shrinkage
Source: PLoS Comput Biol. 2015 Feb 18;11(2):e1004099. doi: 10.1371/journal.pcbi.1004099 (PMC4333834; doi:10.1371/journal.pcbi.1004099)
Supplement: S2 Text — This text describes the methodology for umbrella sampling simulations. (PDF) [file pcbi.1004099.s002.pdf]

Text S2. Monte-Carlo simulations using umbrella sampling:

To obtain the free energy landscape (or probability distribution) for our order parameter R_x, we employ a two step method. In the first step, we calculate the "weights" ($w(R_x)$) by using a modified energy paving method [1]. In the second step, we use these weights to compute the free energy landscape (equivalently, probability distribution function) by the umbrella-sampling method described by Duijneveldt and Frenkel (1992) [2]. Below we will describe these steps.

Weight calculation: The aim of this step is to obtain the weights ($w(R_x)$) such that $F(R_x) + w(R_x)$ will give us a flat energy scape. To do this, we perform multiple cycles of the Metropolis Monte-Carlo simulation with the following rules: First we start with flat weight function $W(R_x, t)$ and after each trial, the functions get modified by $W(R_x, t) = W(R_x, t-1) + j_i$ where j_i is a small number. At the same time, the energy gets modified by

$$\epsilon(R_x, t) = E^{tot}(R_x) + W(R_x, t), \quad \text{(SEq. 3)}$$

where ϵ is the energy of the system. This is continued until all the discretized R_x space is visited at least once (when all the R_x space is visited once, we call it a cycle). At this point $F(R_x) + \epsilon(R_x, t)$ is 'flatter'. Then we repeat the same procedure in the next cycle starting with the current $W(R_x, t)$ and using half of the j value used in the previous cycle, and starting from the straight initial conformation. This will yield a more 'flatter' $\epsilon(R_x, t)$. This procedure is repeated until j in the nth cycle, $j_n \leq 0.00001 k_{\rm B}T$ and we get our weights $w(R_x)$ as $W_n(R_x, t)$ at the end of the nth cycle.

Umbrella sampling: In this step we compute the probability distribution using the umbrella sampling method as described in ref. [2]. Briefly, we perform the Monte-Carlo simulation in the modified landscape such that the Boltzmann weights of the conformations are now given by

$$\exp(-\beta E(R_x(\theta_0, \theta_1, ..., \theta_n)) + \beta \ln w(R_x)) \quad \text{(SEq. 4)}$$

where $w(R_x)$ is the weight function obtained in the previous step. The probability distribution is then computed using $P(R_x) = \frac{\langle \delta(R_x - R_x(\theta_0,\theta_1,...,\theta_n))/w \rangle}{\langle 1/w \rangle}$, where the angular brackets represent thermal average in the modified landscape.

References

[1] Ulrich H E Hansmann and Luc T Wille. *Global optimization by energy landscape paving.* Physical Review Letters **88** (6), 068105 (January 2002).

[2] J S Van Duijneveldt and D Frenkel. *Computer simulation study of free energy barriers in crystal nucleation.* The Journal of Chemical Physics **96** (6), 4655–4668 (1992).
