# Supplementary material for: Statistical Mechanics Provides Novel Insights into Microtubule Stability and Mechanism of Shrinkage
Source: PLoS Comput Biol. 2015 Feb 18;11(2):e1004099. doi: 10.1371/journal.pcbi.1004099 (PMC4333834; doi:10.1371/journal.pcbi.1004099)
Supplement: S3 Text — (PDF) [file pcbi.1004099.s003.pdf]

Text S3. Geometric relationship between R_x and L_c

In the absence of thermal fluctuations, the peeled-off segment of the protofilament assumes a circular conformation which minimizes the bending energy of the L_c segment (See Fig. S1(a)). Given that the radius of the circular arc is $r_c = b/\theta^D$ and the angle shown in the figure is $Q = L_c/r_c$, we get the relation $R_x = L_c - r_c \sin(L_c/r_c)$ (Eq. 7 in main text).
