# Supplementary material for: Statistical Mechanics Provides Novel Insights into Microtubule Stability and Mechanism of Shrinkage
Source: PLoS Comput Biol. 2015 Feb 18;11(2):e1004099. doi: 10.1371/journal.pcbi.1004099 (PMC4333834; doi:10.1371/journal.pcbi.1004099)
Supplement: S4 Text — This text describes the methodology of Langevin dynamics simulation, to incorporate non-equilibrium dissociation of tubulin subunit, to calculate shrinkage velocity. (PDF) [file pcbi.1004099.s004.pdf]

Text S4. Shrinkage velocity calculation using Langevin dynamics simulations

As the MT protofilament tip moves along the free energy landscape, dissociation events can happen stochastically with a rate $w_D = 1/t_{off}$. The evolution of the tip position ($R_x(t)$), as a result of dissociation as well as movement in the landscape (unzippering) can be computed using the procedure discussed below. The evolution of the tip position of the protofilament, as a result of movement in the landscape can be computed using the Langevin equation

$$\tilde{R}_x(n+1) = \tilde{R}_x(n) - \tilde{\mu}\frac{\partial F(R_x)}{\partial R_x} + \sqrt{2\tilde{\mu}}\tilde{\zeta}, \qquad \text{(SEq. 5)}$$

where $\tilde{R}_x = R_x/\lambda$ is the dimensionless position of the tip at the nth 'step' and $\lambda =$ 1nm. $\tilde{\mu} = (\delta t \mu k_{\mathrm{B}} T)/\lambda^2$ is the rescaled mobility and $\tilde{\zeta}$ is the rescaled random force with appropriate noise statistics [1]. Here μ is the effective mobility of the tip as discussed above and δt is the time step. At every time step, the tip subunit could also dissociate from the filament with a probability [2, 3]

$$P(w_D, \tau_D < \delta t) = 1 - e^{-w_D \delta t}. \qquad \text{(SEq. 6)}$$

This dissociation can change the tip position such that $R_x(n+1) = R_x(n) - g$, where g is the projection of terminal subunit of the protofilament (see Fig. S1(a), $g = R_x - R_{x'}$). This dissociation can be thought of as a non-equilibrium jump (one-way switching) from one free energy landscape to another, with similar shape but of shorter length. Note that R_x at any instant is the tip position measured from the straight conformation of the protofilament, having a particular length at that instant. This dynamics of R_x and depolymerization together will result in the length change of the microtubule. Knowing the $R_x(t)$, at any instant, the observed length of microtubule ($\mathcal{L}$) can be computed as (see Fig. S1(b))

$$\mathcal{L}(t) = \mathcal{L}(0) - R_x(t) l_d - b N_d(t), \qquad \text{(SEq. 7)}$$

where l_d is another geometric factor that relates R_x and length (see Fig. S1(b)) and $N_d(t)(= \int_0^t w_D dt')$ is the number of dissociated subunits till time t. We extended the free energy landscape seen by a protofilament to 2000nm by repeating the periodic stretch of the landscape (see Fig. S1(d)) and calculated 100 time-trajectory for various value of ΔE. We choose $w_D = 80$ subunits/s per protofilament as the dissociation rate. Also, in these simulations we assume that the MT subunits do not dissociate when the PF is in the tubular state ($R_x(t) \leq 10$ nm) – i.e, when the lateral bonds are intact.

References

[1] R. Netz. *Nonequilibrium Unfolding of Polyelectrolyte Condensates in Electric Fields.* Physical Review Letters **90** (12), 1–4 (March 2003).

[2] N G Van Kampen. *Stochastic Processes in Physics and Chemistry.* North-Holland Personal Library. Elsevier Science 2011.

[3] Kunkun Guo, Julian Shillcock and Reinhard Lipowsky. *Self-assembly of actin monomers into long filaments: Brownian dynamics simulations.* The Journal of Chemical Physics **131** (1), 015102 (July 2009).
