# Supplementary material for: Statistical Mechanics Provides Novel Insights into Microtubule Stability and Mechanism of Shrinkage
Source: PLoS Comput Biol. 2015 Feb 18;11(2):e1004099. doi: 10.1371/journal.pcbi.1004099 (PMC4333834; doi:10.1371/journal.pcbi.1004099)
Supplement: S5 Text — In this text we derive a simple theory to gain some analytic understanding of occurrence of multiple minima in the free energy. (PDF) [file pcbi.1004099.s005.pdf]

Text S5. Analytical understanding of multiple minima in the free energy

We can qualitatively understand the presence of multiple minima in the free energy landscape $F(R_x)$ with a simple statistical mechanics calculation. To do so we first revisit the model explained in Fig. 1 of the main paper. We simplify the model by assuming that the lateral bonds are either "on" or "off"—when the protofilament is bound to the substrate the favorable energy of lateral interaction per bond is taken as E_m^s, as shown in the main paper. The bending interaction energy between any two subsequent subunits is taken as $k^b/2(\phi_{i+1} - \phi_i - \theta^D)^2$, which is the Taylor series expansion of Eq. 3 in the main text, and is used for computational simplicity; here ϕ_i is the angle of the ith subunit with respect to the horizontal and $\phi_{i+1} - \phi_i = \theta_i$. Note that the quantity $1/2k^b\theta^{D^2}$ is the bending strain energy stored in the GDP-protofilament when they are confined in the straight conformation (E_b^s). If we further assume that the unzippering of the protofilament from the substrate is sequential, then the total energy of the protofilament when $N_c(= L_c/b)$ number of subunits are peeled off is:

$$E^{\mathrm{tot}} = \Delta E N_c + \sum_{i=1}^{N_c} \frac{k^b}{2}(\phi_{i+1} - \phi_i - \theta^D)^2. \tag{SEq. 8}$$

Partition function for the model with no constraint: First, let us calculate the partition function for this model with no constraints. The partition function is given by

$$\mathcal{Z}(N_c) = \underbrace{\int \cdots \int}_{N_c} \exp[-\beta E^{\mathrm{tot}}(N_c)] d\phi_1 \ldots d\phi_{N_c}, \tag{SEq. 9}$$

and evaluates out to

$$\mathcal{Z}(N_c) = \exp(-\beta \Delta E N_c) \left(\sqrt{\frac{2\pi}{k^b \beta}}) \right)^{N_c}, \tag{SEq. 10}$$

$$= \exp\left(\beta N_c(-\Delta E + k_{\mathrm{B}}T \ln \sqrt{\frac{2\pi k_{\mathrm{B}}T}{k^b}}) \right), \tag{SEq. 11}$$

where $\beta = 1/k_{\mathrm{B}}T$ as per standard convention in statistical physics. This first term of the above expression provides the contribution coming the power-struggle between lateral and bending energy whereas, the second term provides the contribution to the free energy from the thermal fluctuations of the peeled off portion of the protofilament. This equation clearly demonstrates that the main term contribution to the partition function, and hence free energy, of the protofilament comes from ΔE since the second term scales as $\ln k^b$, and hence does not strongly depend on the modulus of the bending interactions.

Calculation of Probability distribution function $P(R_x)$ and Free energy $F(R_x)$
The probability distribution function is defined as

$$P(R_x) = \mathcal{A} \sum_{N_c} \underbrace{\int \dots \int}_{N_c} \exp\left[-\beta E^{\text{tot}}\right] \delta \left(R_x - b \sum_{i=1}^{N_c} \cos\phi_i \right) d\phi_1 \dots d\phi_{N_c}, \qquad \text{(SEq. 12)}$$

where the Dirac delta function is to ensure that one is counting only those conformations having the tip position R_x, and $\mathcal{A}$ is the normalization constant. It can be seen that

$$P(R_x) = \mathcal{A} \sum_{N_c} \exp\left[-\Delta E N_c\right] \mathcal{P}(R_x, N_c) \qquad \text{(SEq. 13)}$$

where

$$\mathcal{P}(R_x, N_c) = \mathcal{A} \underbrace{\int \dots \int}_{N_c} \exp\left[-\beta \sum_{i=1}^{N_c} \frac{k^b}{2} (\phi_{i+1} - \phi_i - \theta_D)^2\right] \delta \left(R_x - b \sum_{i=1}^{N_c} \cos\phi_i \right) d\phi_1 \dots d\phi_{N_c} \qquad \text{(SEq. 14)}$$

is the probability distribution function for a semiflexible polymer of length $L_c(= N_c b)$ with intrinsic curvature and no lateral interaction. Given the high bending stiffness of protofilaments, one can reasonably assume that $\mathcal{P}(R_x, N_c)$ will be a highly peaked function around zero temperature value of R_x given by $R_x^0 = L_c - r_c \sin(L_c/r_c)$. Further assuming

$$\mathcal{P}(R_x, N_c) \sim \exp \frac{(R_x - R_x^0)^2}{2\sigma^2} \qquad \text{(SEq. 15)}$$

and substituting this in SEq. 13, we can calculate $P(R_x)$ by summing over N_c numerically. The result for $F(R_x) = -k_B T \ln P(R_x)$ is shown in Fig. S1(c) for a constant $\sigma = 7.07$ and $\Delta E = 0$. Interestingly, this simple calculation captures the main features of the free energy seen in Fig. 2(B) of the main manuscript.
