# Supplementary material for: Statistical Mechanics Provides Novel Insights into Microtubule Stability and Mechanism of Shrinkage
Source: PLoS Comput Biol. 2015 Feb 18;11(2):e1004099. doi: 10.1371/journal.pcbi.1004099 (PMC4333834; doi:10.1371/journal.pcbi.1004099)
Supplement: S6 Text — In this text we discuss the effect of geometric coupling between the bending of protofilament and stretching of lateral interactions in multi-protofilament model and its effect on our results. (PDF) [file pcbi.1004099.s006.pdf]

Text S6. Coupling between bending and stretching of lateral bonds

When a protofilament bends, it also stretches the lateral bonds. From the geometry, one may find that the coupling between this bending and stretching of lateral bonds can depend on the number of protofilaments in the model (or equivalently, the radius of curvature of the microtubule cylinder). Since we use a 3 protofilament model here, it is legitimate to ask how our results will get modified if one uses a full 13 protofilament model. To examine the effect of this geometry-related coupling issue, we did the following. Since a 13-protofilament (PF) system is the reality, we take that as the reference configuration. Let us assume that $k^s_{(13)}$ (lateral interaction stiffness) and $l^{(13)}_{\max}$ (maximum stretching beyond which the bond is broken) are the "real" parameters of the 13-PF MT. The bend-stretch coupling will increase as the number of protofilaments decrease. This implies that if we want to have the same energy for a 3-PF and 13-PF systems, the 3-PF system will have different effective parameters ($k^s_{(3)}$ and $l^{(3)}_{\max}$) that determine the stretching energy of the 3-PF system. A simple way to estimate these effective parameters is the following: Consider the two constraints that are needed to equate the 3PF and 13 PF systems. First, the lateral interaction energy E^s_m should be the same for both 13 and 3 PF systems. i.e., $\frac{1}{2}k^s_{(13)}(\Delta l^{(13)}_{\max})^2 = \frac{1}{2}k^s_{(3)}(\Delta l^{(3)}_{\max})^2 = E^s_m$, where $\Delta l_{\max} = (l_{max} - l^o)$. Additionally, to ensure that the same curved conformation of a PF leads to unzippering in both 3-PF and 13-PF systems, the geometry should be such that $\Delta l^{(13)}_{\max}/\sin(\pi/13) = \Delta l^{(3)}_{\max}/\sin(\pi/3)$. With this simple scheme, we can bring about an equivalence in the lateral and radial coupling between 3 and 13 protofilament models, to a large extent. This tells us that we should make sure that our results are robust for a set of effective parameters based on the above calculation. It is reasonable to assume that the range of lateral interaction in MT ($l^{(13)}_{\max}$) will be in the region of ∼angstroms to nanometer. For a given E^s_m, we took different $l^{(13)}_{\max}$ values in this range and estimated the corresponding k^s and l_{max} for 3 protofilaments. Since multi-protofilament simulations are highly time consuming, we performed our equivalent one-protofilament simulations for a range k^s and l_{max} values and found that our conclusions remain the same. In Fig. S7 we show the $\langle R_x \rangle$ and unzippering velocity for a range of k^s and l_{max} values such that $E^s_m = (1/2)k^s(l_{max} - l^o)^2 = 8k_\mathrm{B}T$ is fixed.
