# Supplementary material for: Statistical Mechanics Provides Novel Insights into Microtubule Stability and Mechanism of Shrinkage
Source: PLoS Comput Biol. 2015 Feb 18;11(2):e1004099. doi: 10.1371/journal.pcbi.1004099 (PMC4333834; doi:10.1371/journal.pcbi.1004099)
Supplement: S1 Fig — The figure show geometric relation between R x and L c, result of theoretical analysis and an example of extended free energy landscape used in Langevin dynamics simulations. (PDF) [file pcbi.1004099.s007.pdf]

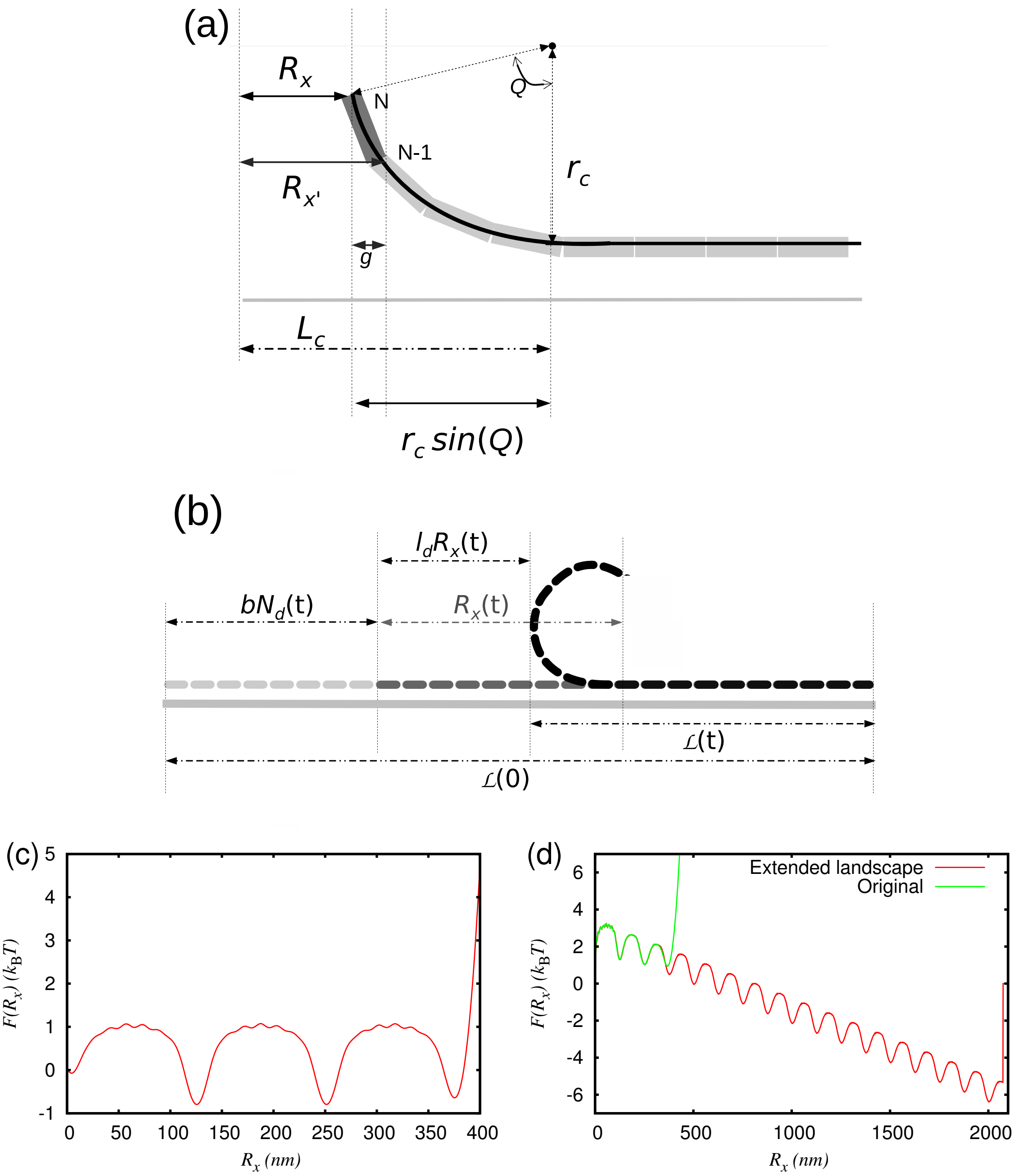

Fig. S1. (a)The figure depicts the geometric relationship between R_x and peeled-off length L_c (Also see Text S3 and S4). (b) The relationship between the initial length of a MT protofilament ($\mathcal{L}(0)$) and the length at time t ($\mathcal{L}(t)$) (Also see Text S4). (c)The free energy calculated using a simple statistical mechanics calculation as described in Text S5. (d) An example of extended free energy landscape ($\Delta E = 1.2k_{\mathrm{B}}T$). See Text S4.
