# Supplementary material for: Statistical Mechanics Provides Novel Insights into Microtubule Stability and Mechanism of Shrinkage
Source: PLoS Comput Biol. 2015 Feb 18;11(2):e1004099. doi: 10.1371/journal.pcbi.1004099 (PMC4333834; doi:10.1371/journal.pcbi.1004099)
Supplement: S2 Fig — (PDF) [file pcbi.1004099.s008.pdf]

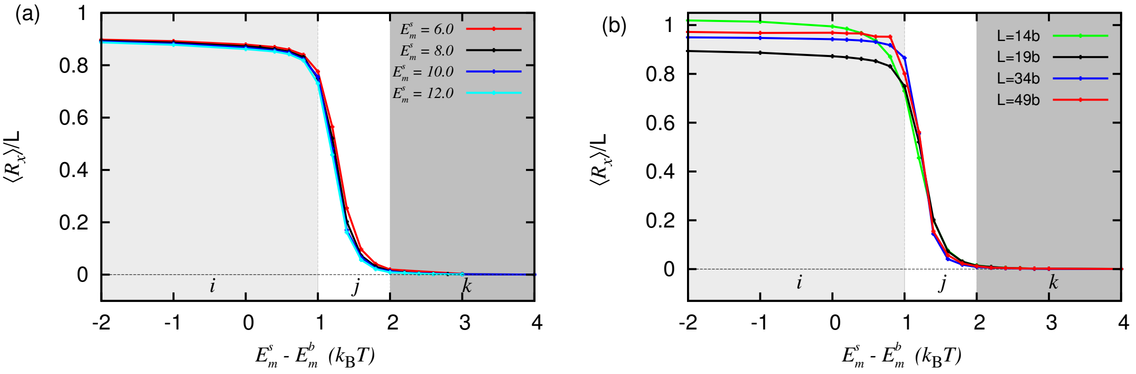

Fig. S 2. The results shown in Fig. 2 of main text are similar for a wide range of parameters.

Average x position of the tip $\langle R_x \rangle$ of a GDP protofilament as a function of the power-struggle parameter $E_m^s - E_m^b = \Delta E$, in the single protofilament version of the model. (a) $\langle R_x \rangle$ with different E_m^s parameters. For each value of E_m^s, $\langle R_x \rangle$ is computed by varying E_m^b. These simulations are performed for $L = 19b$. Notice that all the curves have same behavior for different value of E_m^s. (b)$\langle R_x \rangle$ for various values of L, keeping $E_m^s = 8k_\mathrm{B}T$. This plot suggests that the overall behavior of the system, for various L values are not significantly different.
