# Supplementary material for: Statistical Mechanics Provides Novel Insights into Microtubule Stability and Mechanism of Shrinkage
Source: PLoS Comput Biol. 2015 Feb 18;11(2):e1004099. doi: 10.1371/journal.pcbi.1004099 (PMC4333834; doi:10.1371/journal.pcbi.1004099)
Supplement: S3 Fig — (PDF) [file pcbi.1004099.s009.pdf]

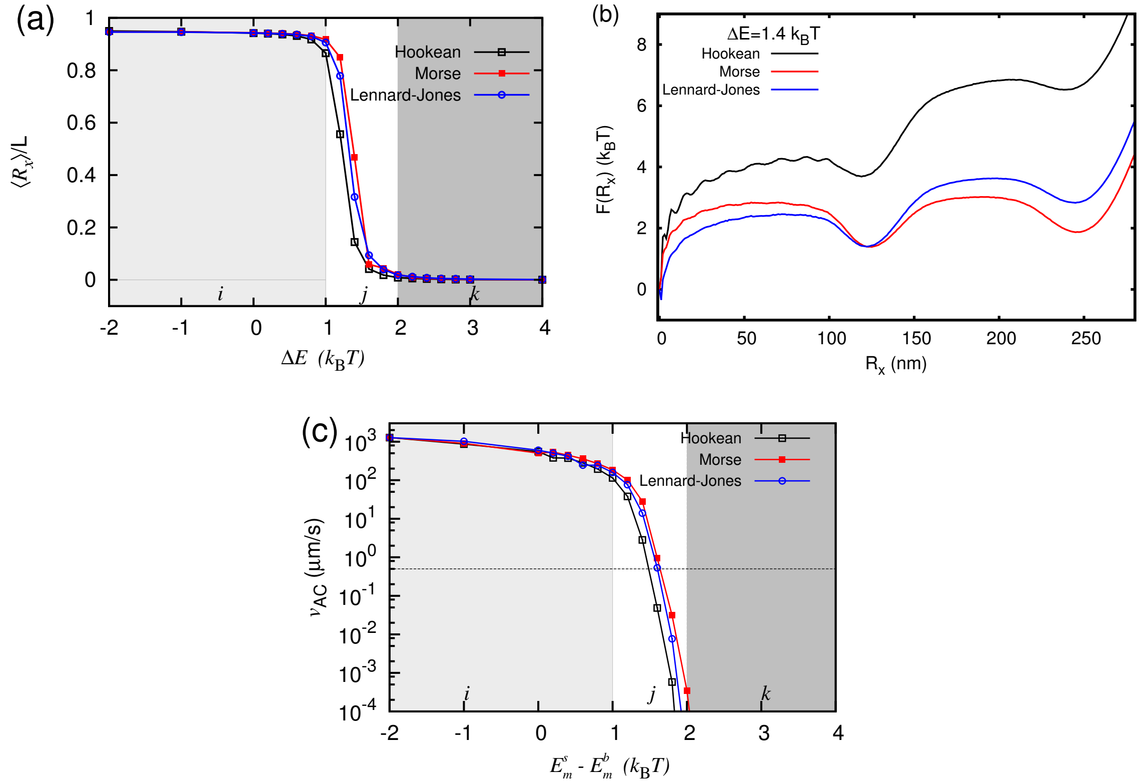

Fig. S 3. Alternative energy functions for lateral interactions. In the main manuscript we have described lateral interactions using breakable Hookean spring (Eq. 3). To understand how the nature of lateral energy function affect our main results, we have also performed simulations with Lennard-Jones potential ($E_i^{s,LJ} = E_m^s((l^o/l_i)^{12} - 2(l^o/l_i)^6)$, for $E_m^s = 8.0k_{\rm B}T$ and $l_o = 6.5$nm), and Morse potential ($E_i^{s,M} = E_m^s(1 - e^{-\delta(l_i - l^o)^2})$, for $E_m^s = 8.0k_{\rm B}T$, $l_o = 6.5$nm and $\delta = 1$). See Text S1. Similar to the case of Hookean spring (main text), we varied ΔE by varying k^b and computed $\langle R_x \rangle$, $F(R_x)$ and v_{AC} as done in the main text. Figure (a), (b) and (c) correspond to the Fig. 2(a), Fig. 2(b) and Fig. 3(a) of the main manuscript, respectively. From the figure it is evident that the nature of the potential does not alter the main findings of the study.
