# Supplementary material for: Statistical Mechanics Provides Novel Insights into Microtubule Stability and Mechanism of Shrinkage
Source: PLoS Comput Biol. 2015 Feb 18;11(2):e1004099. doi: 10.1371/journal.pcbi.1004099 (PMC4333834; doi:10.1371/journal.pcbi.1004099)
Supplement: S4 Fig — (PDF) [file pcbi.1004099.s010.pdf]

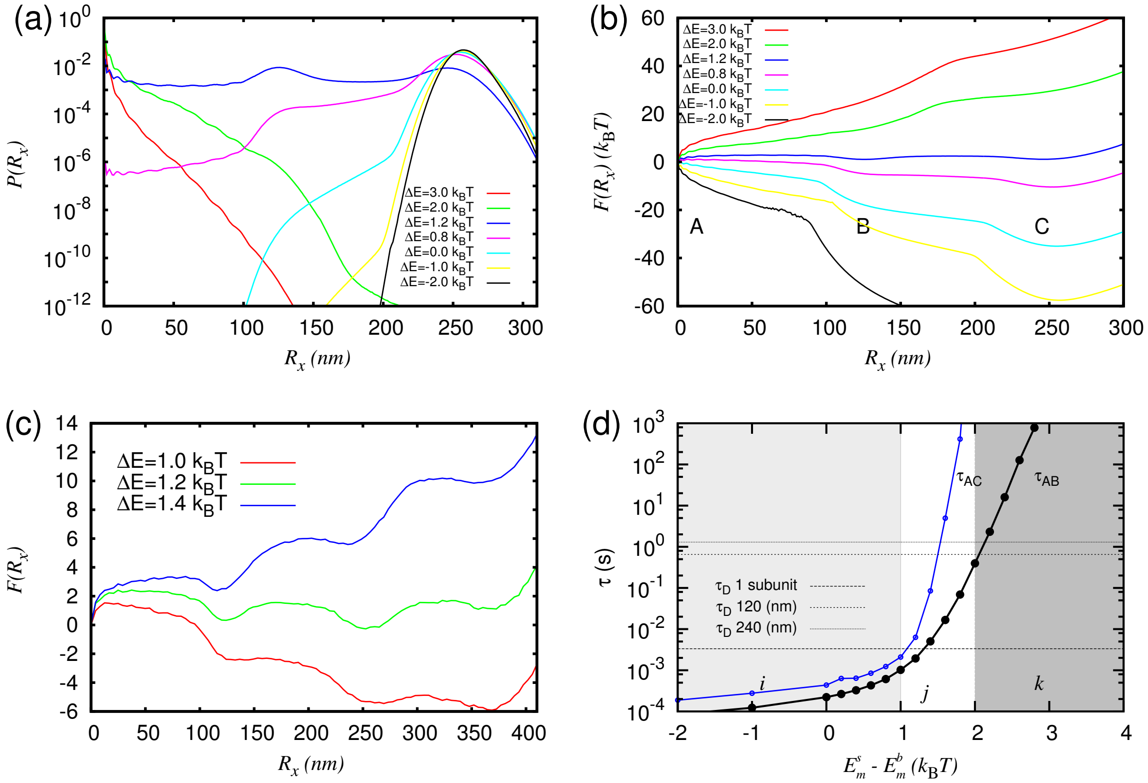

Fig. S 4. $P(R_x)$, $F(R_x)$, **and unzippering timescales.** (a) and (b) are $P(R_x)$ and $F(R_x)$, respectively, for various value of ΔE, where E^s_m is kept fixed at $8.0k_\mathrm{B}T$ and $L = 34b$. (c) Multiple minima in the free energy can be seen at all values of $R_x = n2\pi r_c$ (see main text) even for a larger length of $L = 49b$. Here the free energy is computed using the pavement method as in Text S2. (d) Unzippering timescale from state A to state B (τ_{AB}) and from state A to state C (τ_{AC}) for various value of ΔE; here E^m_s is kept fixed at $8.0k_\mathrm{B}T$ and $L = 34b$.
