# Supplementary material for: Statistical Mechanics Provides Novel Insights into Microtubule Stability and Mechanism of Shrinkage
Source: PLoS Comput Biol. 2015 Feb 18;11(2):e1004099. doi: 10.1371/journal.pcbi.1004099 (PMC4333834; doi:10.1371/journal.pcbi.1004099)
Supplement: S5 Fig — (PDF) [file pcbi.1004099.s011.pdf]

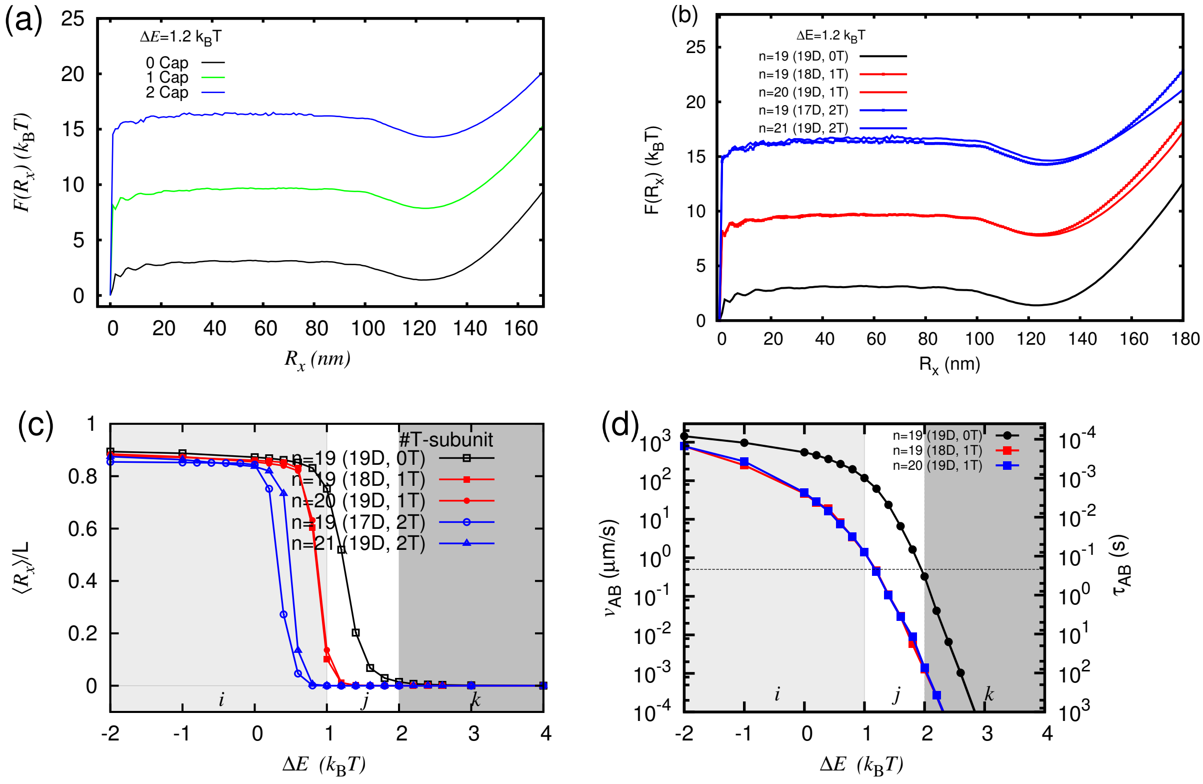

Fig. S5. The influence of GTP-cap on the stability of microtubules. (a) Free energy ($F(R_x)$) profile for $L = 19b$ at $\Delta E = 1.2 k_{\mathrm{B}}T$ for different T-cap sizes. Note that the barrier at $R_x = 0$ increases with the cap size. In these simulations, the total length of the protofilament remains a constant (number of T-subunits + number of D-subunits = 19). Figs. (b), (c) and (d) show the effect of GTP-cap in an alternative ensemble, where the number of D-subunits is kept constant; the total length of protofilament for no cap is $19b$, for one layer of cap is $20b$ and for two layers of cap is $21b$. (b) , (c) and (d) show the comparison of various results obtained between these two different ensembles.
