# Supplementary material for: Statistical Mechanics Provides Novel Insights into Microtubule Stability and Mechanism of Shrinkage
Source: PLoS Comput Biol. 2015 Feb 18;11(2):e1004099. doi: 10.1371/journal.pcbi.1004099 (PMC4333834; doi:10.1371/journal.pcbi.1004099)
Supplement: S6 Fig — (PDF) [file pcbi.1004099.s012.pdf]

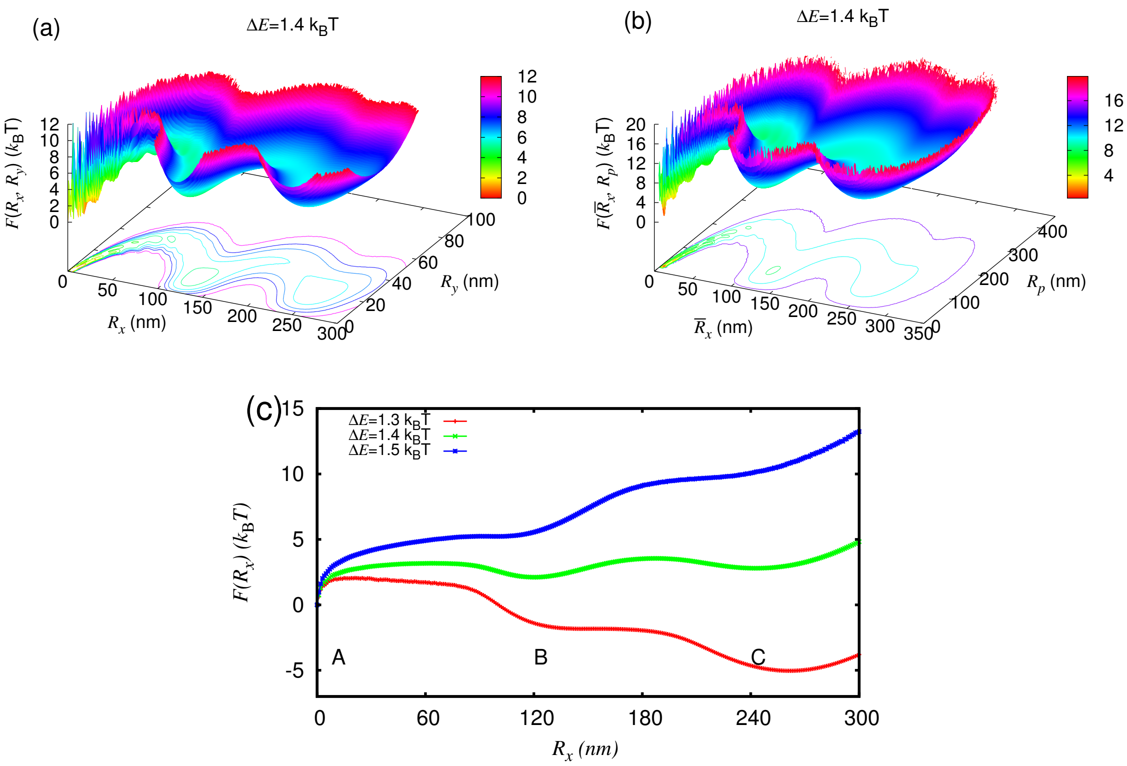

Fig. S6. Free energy from the multi-protofilament model. (a) Free energy as a function of the x position (R_x) and y position (R_y) of the tip of a single protofilament in the multi-protofilament model. Similar to the 1D case, the free energy in 2D shows multiple minima at $R_x = 0, = 120$nm and $= 240$nm. Note that the R_y values are bounded in a small range and the free energy, in the R_y direction, increases sharply for any change in R_y from its minimum. (b) Free energy as a function of $\bar{R_x}$ and R_p, where $\bar{R_x} = (R_{x,1} + R_{x,2} + R_{x,3})/3$ is the average x tip position of the three protofilaments and R_p is the perimeter of the triangle formed out of the three tip positions. In this landscape $\bar{R_x}$ and R_p behave similar to R_x and R_y in the previous figure. $\bar{R_x}$ has multiple minima at $\bar{R_x} = 0, = 120$nm and $= 240$nm. As seen in the Figure R_p behaves similar to R_y as expected, since there is some geometrical correlation between R_p and R_y. However, unlike the previous figure, this contains the cumulative behavior of all the three protofilaments. (c) Free energy distribution ($F(R_x)$) of a single protofilament in the multi-protofilament model for different values of ΔE. These simulations are performed for $L = 35b$, $E_m^s = 4k_BT$ in the 3-protofilament system ($p = 3$). The distributions are calculated using the Metropolis Monte-carlo method ($\approx 10^{10}$ Monte Carlo steps).
