# Supplementary material for: Statistical Mechanics Provides Novel Insights into Microtubule Stability and Mechanism of Shrinkage
Source: PLoS Comput Biol. 2015 Feb 18;11(2):e1004099. doi: 10.1371/journal.pcbi.1004099 (PMC4333834; doi:10.1371/journal.pcbi.1004099)
Supplement: S7 Fig — (PDF) [file pcbi.1004099.s013.pdf]

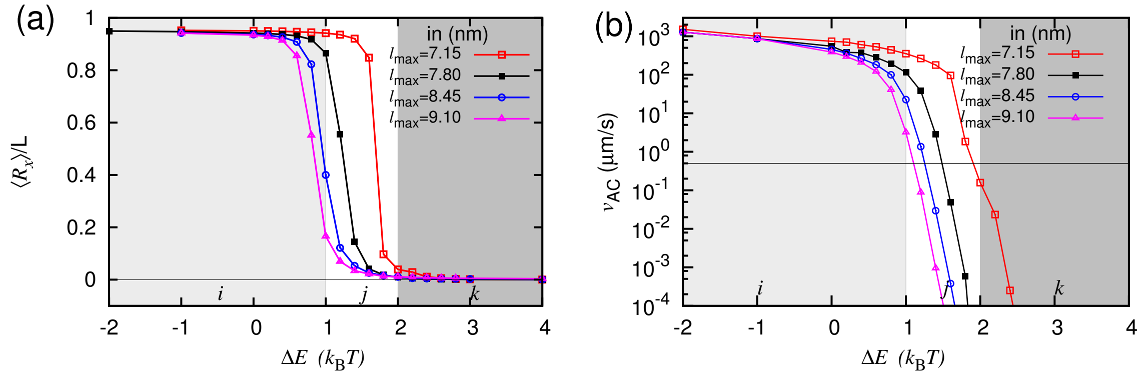

Fig. S7. The main results in this manuscript are robust for a wide range of k^s and l_{max} values: (a)$\langle R_x \rangle$ and (b) unzippering velocity as a function of ΔE for different k^s and l_{max} values such that $E_m^s = (1/2)k^s(l_{max} - l^o)^2 = 8k_{\rm B}T$ is fixed. Given that the main conclusions from all of these curves are similar for a wide range of effective parameters, one expects that "real" parameters of microtubules (within a corresponding 13-protofilament model) will fall in this range and the results will be similar. (See Text S6).
